# Identification of tumor suppressor miRNAs by integrative miRNA and mRNA sequencing of matched tumor–normal samples in lung adenocarcinoma

**DOI:** 10.1002/1878-0261.12478

**Published:** 2019-04-18

**Authors:** Namhee Yu, Seunghui Yong, Hong Kwan Kim, Yoon‐La Choi, Yeonjoo Jung, Doyeon Kim, Jihae Seo, Ye Eun Lee, Daehyun Baek, Jinseon Lee, Seungjae Lee, Jong Eun Lee, Jaesang Kim, Jhingook Kim, Sanghyuk Lee

**Affiliations:** ^1^ Department of Life Science Ewha Womans University Seoul Korea; ^2^ Ewha Research Center for Systems Biology (ERCSB) Ewha Womans University Seoul Korea; ^3^ Department of Thoracic and Cardiovascular Surgery Samsung Medical Center Sungkyunkwan University School of Medicine Seoul Korea; ^4^ Department of Pathology and Translational Genomics Samsung Medical Center Sungkyunkwan University School of Medicine Seoul Korea; ^5^ Center for RNA Research Institute for Basic Science Seoul Korea; ^6^ School of Biological Sciences Seoul National University Korea; ^7^ Samsung Biomedical Research Institute Samsung Medical Center Sungkyunkwan University School of Medicine Seoul Korea; ^8^ DNA Link Inc. Seoul Korea

**Keywords:** biomarker, lung adenocarcinoma, miRNA, transcriptome analysis

## Abstract

The roles of miRNAs in lung cancer have not yet been explored systematically at the genome scale despite their important regulatory functions. Here, we report an integrative analysis of miRNA and mRNA sequencing data for matched tumor–normal samples from 109 Korean female patients with non‐small‐cell lung adenocarcinoma (LUAD). We produced miRNA sequencing (miRNA‐Seq) and RNA‐Seq data for 48 patients and RNA‐Seq data for 61 additional patients. Subsequent differential expression analysis with stringent criteria yielded 44 miRNAs and 2322 genes. Integrative gene set analysis of the differentially expressed miRNAs and genes using miRNA–target information revealed several regulatory processes related to the cell cycle that were targeted by tumor suppressor miRNAs (TSmiR). We performed colony formation assays in A549 and NCI‐H460 cell lines to test the tumor‐suppressive activity of downregulated miRNAs in cancer and identified 7 novel TSmiRs (miR‐144‐5p, miR‐218‐1‐3p, miR‐223‐3p, miR‐27a‐5p, miR‐30a‐3p, miR‐30c‐2‐3p, miR‐338‐5p). Two miRNAs, miR‐30a‐3p and miR‐30c‐2‐3p, showed differential survival characteristics in the Tumor Cancer Genome Atlas (TCGA) LUAD patient cohort indicating their prognostic value. Finally, we identified a network cluster of miRNAs and target genes that could be responsible for cell cycle regulation. Our study not only provides a dataset of miRNA as well as mRNA sequencing from the matched tumor–normal samples, but also reports several novel TSmiRs that could potentially be developed into prognostic biomarkers or therapeutic RNA drugs.

**Abbreviations**
DEG: differentially expressed gene
DEmiR: differentially expressed miRNA
LUAD: lung adenocarcinoma
qRT‐PCR: quantitative real‐time PCR
TCGA: Tumor Cancer Genome Atlas
TSmiR: tumor suppressor miRNA

## 1 Introduction

miRNAs are an important class of regulators determining cellular fates in almost all biological processes. A typical miRNA negatively regulates expression of multiple target genes by binding to mRNAs and inhibiting translation or inducing mRNA degradation. A number of miRNAs have been reported to contribute to tumor development, disease progression, and treatment response in nearly all human cancers and have emerged as promising and biologically relevant biomarkers (Kasinski and Slack, 2011).

Most previous studies are based on investigating miRNAs that are predicted to target known cancer‐related pathways, oncogenes, and tumor suppressor genes. For example, the let‐7 miRNA plays a tumor‐suppressive role in lung cancer by targeting RAS and cMYC genes, which are critical regulators of the prominent oncogenic pathway of RAS‐RAF‐MEK‐ERK signaling (He *et al*., 2010; Johnson *et al*., 2005; Kumar *et al*., 2008). On the other hand, the tumor suppressor TP53 gene often described as ‘the guardian of the genome’ is regulated directly and indirectly by multiple miRNAs constituting an intricate regulatory network to mediate the tumor‐suppressive role of p53 (Hermeking, 2012; Liu *et al*., 2017).

Gene expression profiling is a powerful yet unbiased method to identify miRNAs of functional significance. miRNA microarrays, although frequently utilized owing to their cost‐effectiveness, usually suffer from uneven hybridization. This is in large part due to the extremely limited probe design based on the short length of 22 nucleotides in mature miRNAs (Yanaihara *et al*., 2006). Deep sequencing is a potentially ideal method, but the isolation of mature miRNAs and sequencing much shorter reads than in mRNA sequencing are challenging (Ma *et al*., 2014).

A number of miRNAs were implicated in lung adenocarcinoma (LUAD). Analysis of miRNA‐Seq data from the Tumor Cancer Genome Atlas (TCGA) LUAD cohort yielded many differentially expressed miRNAs (DEmiR) with prognostic value including miR‐31, miR‐196b, miR‐101‐1, miR‐187, miR‐331, miR‐375, miR‐519a‐1, miR‐551b, miR‐766, and miR‐3653 (Li *et al*., 2014; Lin *et al*., 2016). However, most of these miRNAs were not validated from independent data sets to be established as reliable prognostic markers. Several miRNAs were additionally implicated to have roles in tumorigenesis of LUAD by targeting known cancer‐related pathways. Examples include miR‐195 targeting CCND3 and BIRC5 (Yu *et al*., 2018), miR‐378 targeting RBX1 and miR‐1827 targeting CRKL (Ho *et al*., 2018), miR‐383‐5p targeting PPP2CA (Zhao *et al*., 2017), miR‐23b and miR125a‐5p targeting KRAS and NF‐kB pathways (Naidu *et al*., 2017). Many of these studies, however, were based on functional assays using cell lines, thus having limited applicability to patients.

Simultaneous profiling of miRNAs and mRNAs provides an opportunity to compare the gene expression of miRNAs and their target mRNAs without extra efforts in filtering out false positives from miRNA–target prediction. Cancer genome projects of the TCGA consortium are good examples, but the portion of patients with both miRNA and mRNA sequenced is limited. Furthermore, sequencing matched tumor–normal samples is important to avoid any individual‐specific biases, but the number of patients with matched sequencing is again rather small especially in the case of miRNA‐Seq. Taking the TCGA LUAD cohort as an example, mRNA sequencing (RNA‐Seq) data are available for 515 tumor and 59 normal samples, including 58 matched pairs. For miRNA‐Seq data, it includes 450 tumor and 46 normal samples, including 39 matched pairs (Cancer Genome Atlas Research, 2014). Only 12 patients have both mRNA‐Seq and miRNA‐Seq data for matched tumor–normal samples. This highlights the difficulties in sample acquisition and sequencing suitable for study designs involving simultaneous profiling of miRNAs and mRNAs.

Here, we performed an integrative analysis on miRNA and mRNA sequencing data for matched tumor–normal samples from 109 LUAD patients and report several tumor suppressor miRNAs (TSmiR) validated from functional experiments. We further identify candidate miRNAs that could be developed into prognostic biomarkers for patient stratification or therapeutic RNA drugs for repressing target oncogenes.

## 2 Materials and methods

### 2.1 miRNA‐Seq and mRNA‐Seq data production

Patient samples were obtained from LUAD patients who had undergone curative surgery in Samsung Medical Center (Seoul, Korea). All samples were collected with the written informed consent from patient and the prior approval of the institutional review board (Samsung Medical Center Institutional Review Board) in accordance with the Declaration of Helsinki.

RNA purity was determined by assaying 1 μL of the total RNA extract on a NanoDrop ND‐1000 spectrophotometer (ThermoFisher, Waltham, MA, USA). Total RNA integrity was checked using a Bioanalyzer 2100 with an RNA Integrity Number value greater than 8 (Agilent, Santa Clara, CA, USA). Then, mRNA sequencing libraries were prepared according to the manufacturer's instructions using the Illumina Truseq RNA Prep kit v2. The quality of the amplified libraries was verified again with an Agilent Bioanalyzer 2100. Sequencing of pooled libraries was performed on the HiSeq 2000 sequencing system with paired‐end reads of 100 bp length (Illumina, San Diego, CA, USA).

Small RNA sequencing libraries were prepared according to the manufacturer's instructions using the Illumina Small RNA Prep kit. cDNA size selection was carried out with the Sage Science's Pippin prep electrophoresis platform. Sequencing of pooled libraries was performed on the HiSeq 2000 sequencing system (Illumina) with single‐end reads of 50 bp length. Deep sequencing data were deposited at the Gene Expression Omnibus (GSE110907).

### 2.2 Transcriptome data processing

The in‐house workflows for analyzing miRNA‐Seq and RNA‐Seq data are illustrated in Fig. S1. For miRNA‐Seq data, sequencing reads had adapter sequences removed and been mapped to the miRBase release 19 (Kozomara and Griffiths‐Jones, 2011) using bowtie V.0.12.9 (Langmead *et al*., 2009) with the perfect match option. The mapping rates ranged from 63% to 70%. miRNA abundance was quantified using the quantile normalization method in R. mRNA‐Seq data were mapped to the NCBI GRCh 37 genome using mapsplice version v2.1.6 and the gene model in Ensembl GRCh 37.72. Transcript abundance was estimated at the gene level by rsem version 1.2.5 (Li and Dewey, 2011). Statistics of mapped reads and mapping rates are summarized in Table S1.

### 2.3 Identification of differentially expressed miRNAs and genes

We developed a stringent pipeline to identify DEmiRs and genes (DEGs), taking advantage of the matched nature of tumor and normal samples (Fig. S2). Three different programs, edger (Version 3.16.5) (Ritchie *et al*., 2015), voom (limma 3.30.13) (Ritchie *et al*., 2015), and deseq2 (Version 1.14.1) (Anders and Huber, 2010), were used to select DEmiRs with false discovery rate (FDR) ≤10^−5^ after initial filtering of lowly expressed miRNAs. Taking common miRNAs from the three program outputs yielded 142 DEmiRs. We further filtered out lowly expressed DEmiRs by requiring an overall average expression level of logCPM (counts per million) ≥3. Then, we further applied two consistency criteria—(a) the direction of up/down regulation between tumor and normal tissues consistent in over 80% of the total patients and (b) the number of patients with over twofold change equaling over 70% of the total patients. As illustrated in Fig. S2, the rigorous filtering procedure predominantly kept commonly predicted miRNAs from the three algorithms.

For mRNA sequencing data, we applied a slightly modified pipeline. Instead of looking at the overall average expression level, we required the overall average fold change to be |logFC| > 0.5. The consistency criteria were each relaxed by 10% to allow for more DEGs. The pipeline and number of DEGs at each step are illustrated in Fig. S2.

### 2.4 Compiling and predicting miRNA–target genes

Two main sources of miRNA–target information were miRGator 3.0 (Cho *et al*., 2013) and the latest TargetScan 7.0 (Garcia *et al*., 2011). miRGator 3.0 is a composite database of miRNA targets encompassing three literature‐based knowledgebases (Tarbase, miRecords, and miRTarBase) and six prediction programs (TargetScan 6.2, PITA, PicTar, miRNA.org, miRDB, and Microcosm Targets). miRNA targets commonly predicted by three or more programs are regarded as reliable and merged into miRNA targets in the knowledgebases. In order to make up for not being up‐to‐date in the miRGator 3.0 content, we further imported the prediction results from the latest TargetScan 7.0 (cumulative weighted context score >0.2 as suggested by the developers). Our final compilation included 248 543 miRNA–target relations covering 687 miRNAs and 16 563 target genes.

For selecting target genes of tumor‐suppressive DEmiRs for validation experiments, we used a new conservative prediction program based on a recent study that explored miRNA–target space extensively (Kim *et al*., 2016). In essence, it calculates the multiple linear regression (MLR) score for a target mRNA by considering four biological contexts (local AU content of the flanking region of the miRNA–target site, 3′UTR length, target‐site abundance, and thermodynamic pairing stability between the miRNA and the target mRNA). Only the 8mer, 7mer‐m8, and 7mer‐A1 site types were considered in regression because the 6mer site type was known to exert relatively weak repression on their target mRNAs and thus can act as noise in target prediction (Bartel, 2009). We selected mRNAs with MLR score <−0.3 as putative targets of DEmiRs for further validation experiments.

### 2.5 Cell culture, transfection, and colony formation assay

Human lung cancer cell lines A549 and NCI‐H460 were obtained from the American Type Culture Collection (Manassas, VA, USA). Cells were maintained at 37 °C and 5% CO_2_ in RPMI 1640 supplemented with 10% fetal bovine serum (Hyclone, Logan, UT, USA) and 1% penicillin/streptomycin (Gibco, Invitrogen Corporation, Grand Island, NY, USA).

miRNA mimics were transfected into 1 × 10^5^ cells in a 35‐mm dish using Lipofectamine RNAiMAX (Invitrogen, Carlsbad, CA, USA) at the concentration of 40 nm for 48 h according to the manufacturer's instructions. Candidate TSmiR mimics and the negative control (NC) mimics were purchased from Ambion (Austin, TX, USA).

Two days after transfection with miRNA or NC mimics, 500 cells were replated in a 35‐mm dish in duplicate and incubated at 37 °C and 5% CO_2_ for 6–8 days. Colonies were stained with 0.1% Coomassie Blue in a 45% methanol and 10% acetic acid solution, and colony numbers were determined using the Gel Doc XR system (Bio‐Rad, Hercules, CA, USA) with the Quantity One® 1‐D analysis software (Bio‐Rad). Each experiment was performed in triplicates.

### 2.6 RNA extraction and quantitative reverse transcriptase–PCR (qRT‐PCR)

Total RNA was extracted from the miRNA mimic‐transfected cells using the miRNeasy Mini kit (Qiagen, Valencia, CA, USA) according to the manufacturer's instructions. Single‐stranded cDNAs were synthesized from 1 μg of the total RNA using the ImProm‐II™ reverse transcriptase (Promega, Madison, WI, USA). For quantitative analysis of miRNA–target mRNA levels, cDNAs generated from 10 ng of the total RNA were subjected to PCR amplification using the CFX96 Real‐time PCR detection system (Bio‐Rad) with the SYBR Select Master Mix (Applied Biosystems by Life Technologies, Austin, TX, USA; Table S2). ACTB and HPRT1, two housekeeping genes, were used as dual reference genes. Cycling conditions were as follows: pre‐denaturation for 2 min at 95 °C, a 2‐step reaction (40 cycles) for 10 s at 95 °C and 40 s at 60 °C, and a dissociation peak analysis. mRNA expression values of target genes were calculated with the Bio‐Rad CFX Manager Software.

## 3 Results

### 3.1 Patient cohort and transcriptome sequencing

Matched tumor–normal samples were obtained from 109 LUAD patients who had undergone curative surgery. All samples were collected with written informed consent from the patients and prior approval of the institutional review board (Samsung Medical Center). All cases were first‐time lung cancer patients and were females. Most patients were never‐smokers except eight smoker cases. Clinicopathological characteristics of what we call ‘ES_Korea’ samples are summarized in Table 1 (with further details provided in Table S3).

**Table 1:** Patient characteristics of the Korean and Tumor Cancer Genome Atlas cohorts

Characteristic	ES_Korea		TCGA_LUAD
	miRNA (*n* = 48)	mRNA (*n* = 109)	miRNA (*n* = 39)
Sex			
Female	48 (100%)	109 (100%)	20 (51%)
Male	–	–	19 (49%)
Age at diagnosis			
Median	59	61.5	66
Range	37–78	29–83	47–85
Stage			
I	30 (63%)	72 (66%)	24 (62%)
II	6 (12%)	15 (14%)	10 (25.5%)
III	12 (25%)	22 (20%)	5 (12.5%)
Ethnicity			
Black or African American	–	–	6 (15%)
White	–	–	33 (85%)
Asian	48 (100%)	109 (100%)	–
Smoking status			
Never‐smoker	41 (85%)	102 (94%)	2 (5%)
Smoker	7 (15%)	7 (6%)	34 (87%)
Unknown	–	–	3 (8%)

We performed both miRNA‐Seq and mRNA‐Seq for the matched tumor–normal samples from 48 individuals. For the remaining 61 individuals, we produced only mRNA‐Seq data for the matched tumor–normal samples. We also downloaded the miRNA‐Seq data from the TCGA LUAD consortium (version 2016_01_28 from the Broad GDAC Firehose), which included 39 cases with sequencing data from both matched tumor–normal samples. Both ES_Korea and the TCGA miRNA‐Seq data sets were processed using our own computational pipeline as described in the Materials and methods section.

### 3.2 Identification of differentially expressed miRNAs and genes

**Figure 1:**
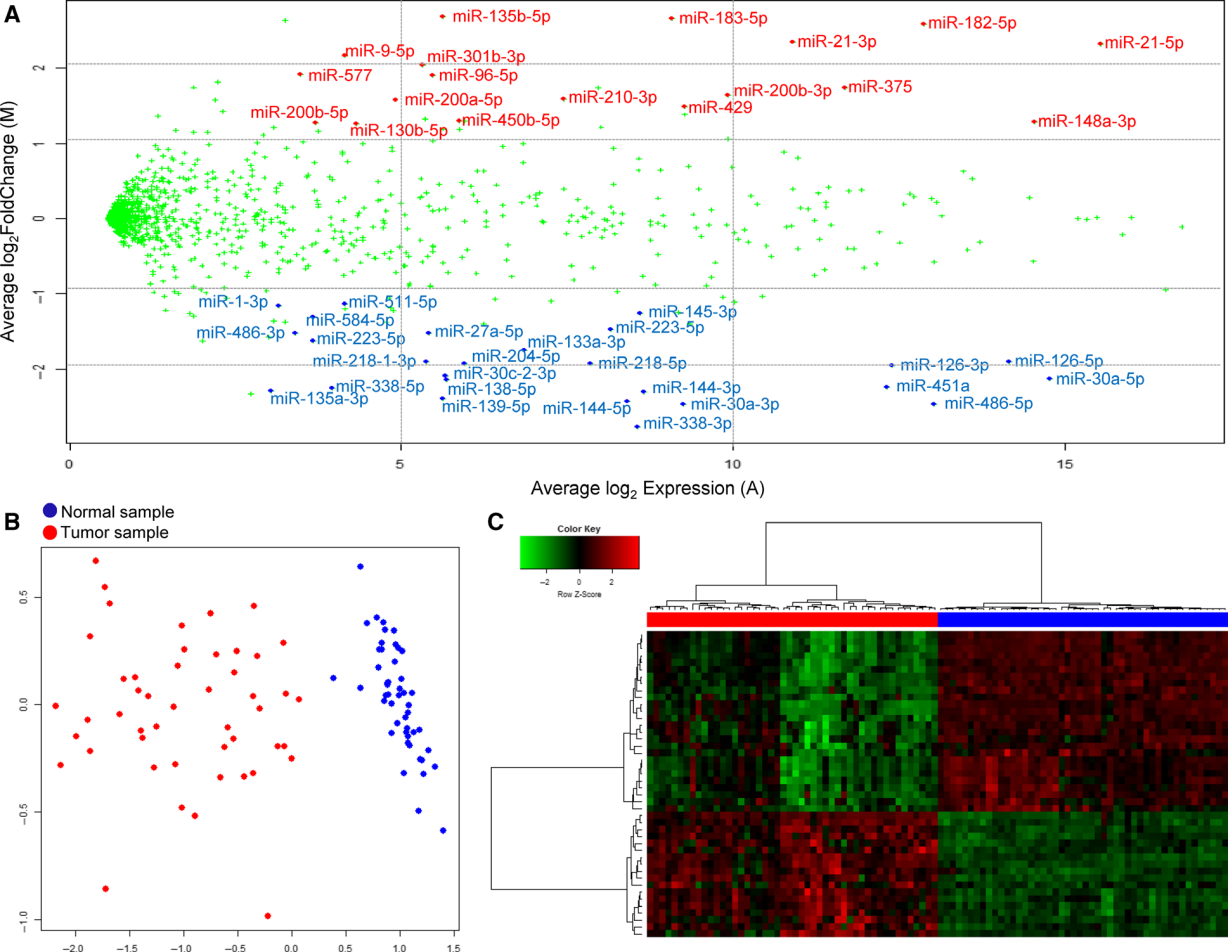
miRNA expression in 48 Korean patients of LUAD. (A) The MA plot where the log fold change (log_2_ exp_tumor/exp_normal) and the average expression (½log_2_ exp_tumor×exp_normal) are shown in the *y*‐axis and *x*‐axis, respectively. Average miRNA expression values over 48 individuals were used in the plot. DEmiRs were indicated in red (upregulated in tumor) and blue (downregulated in tumor) colors. (B) Representation of tumor and normal samples in two‐dimensional space obtained by a multidimensional scaling method. (C) Hierarchical clustering of samples using 44 DEmiRs.

The matched tumor–normal samples enabled us to obtain a robust list of DEmiRs and mRNAs (DEGs). We devised a stringent computational pipeline to predict DEmiRs and DEGs, combining three different programs and rigorous filtering steps such as a consistency criterion that required consistent direction of expression change in more than 80% of the total patients (Fig. S1). Analyzing miRNA‐Seq data from the ES_Korea cases with our pipeline, we obtained 44 highly reliable DEmiRs including 18 up‐ and 26 downregulated miRNAs in tumor (Table S4). The MA plot shows the average fold change vs. the average expression level of these DEmiRs (Fig. 1A). The heatmap and multidimensional scaling views indicate that tumor and normal samples were perfectly classified into two groups according to the expression of these 44 DEmiRs (Fig. 1B,C).

**Figure 2:**
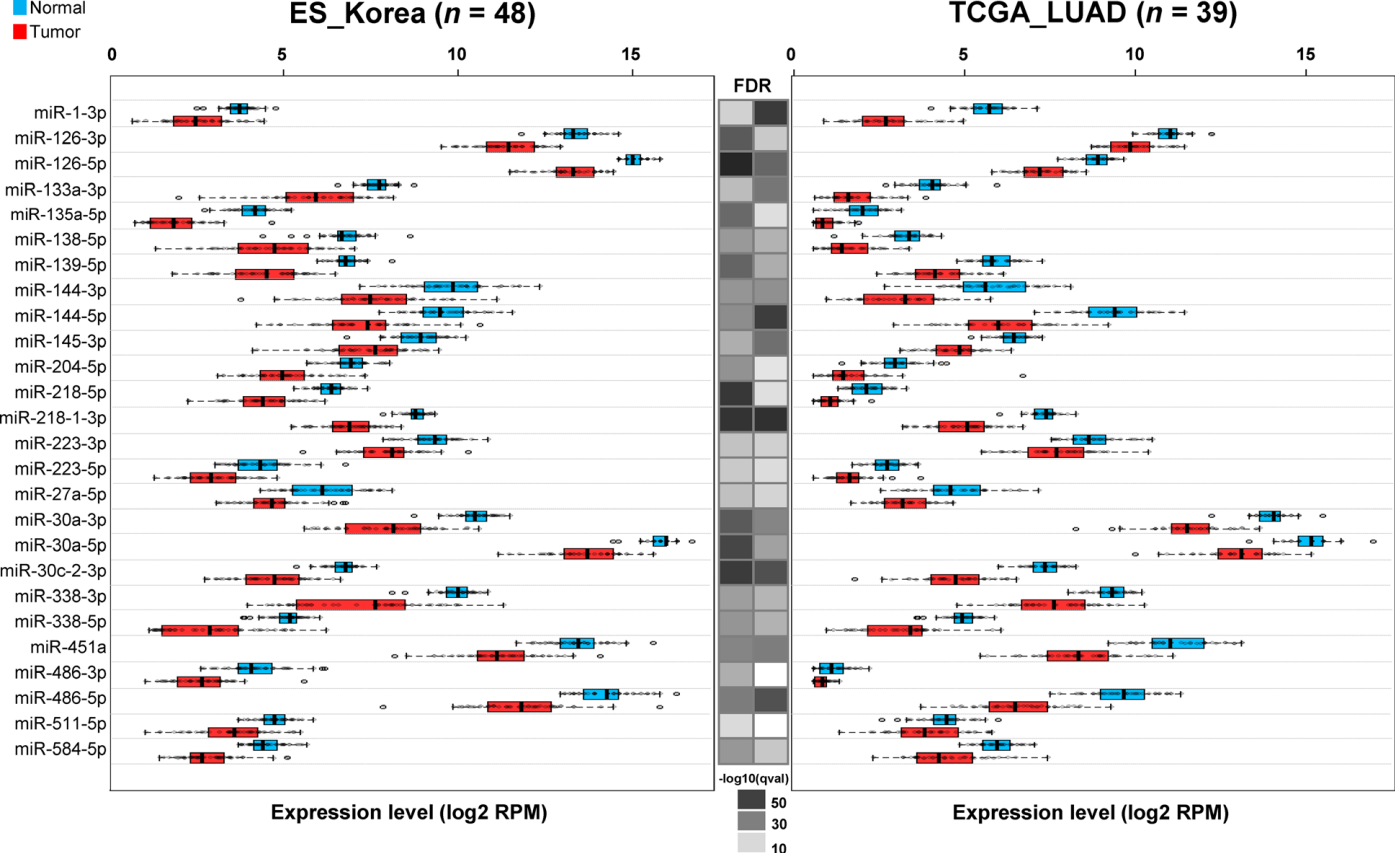
Expression box plots for 26 miRNAs downregulated in tumor samples of the ES_Korea cohort. Tumor and normal samples are indicated in red and blue colors, respectively. miRNA expression in the TCGA cohort (39 patients with the matched tumor–normal samples) is shown on the right for comparison. The heatmap in the middle shows the *q*‐value of the FDR test for differential expression in −log_10_(*q*‐value). Note that box plots for miRNAs upregulated in tumor samples are provided in Fig. S3.

Identical analysis of miRNA‐Seq data from 39 patients of the TCGA LUAD yielded 47 DEmiRs (19 up‐ and 28 downregulated ones), 25 of which overlapped with the ES_Korea DEmiRs. Expression of DEmiRs in tumor and normal tissues is depicted in the box plot (Figs 2 and S3), and we observed that most miRNAs are consistently differentially regulated between ES_Korea and TCGA LUAD data sets except a few cases (miR‐450b‐5p, miR‐486‐3p, and miR‐511‐5p).

Paired RNA‐Seq data were subjected to the computational pipeline for DEGs, where the filtering criteria were alleviated a little bit to prevent missing genuine miRNA targets (Fig. S2). We obtained 2322 DEGs as the final result (935 upregulated and 1387 downregulated genes).

**Figure 3:**
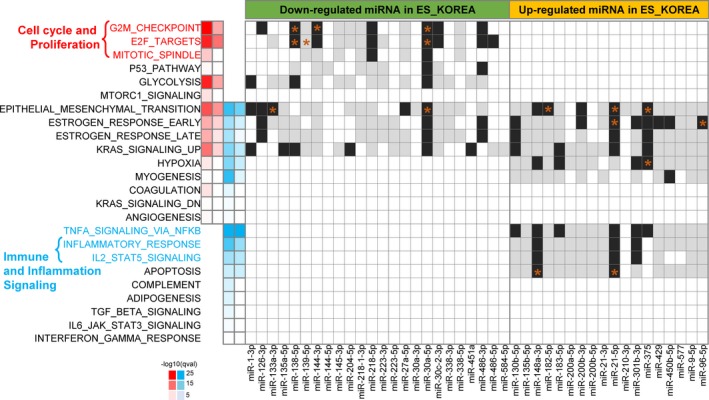
Functional enriched terms of hallmark signatures from MSigDB and relevant miRNAs. The heatmap on the left indicates the *q*‐value of enrichment in −log_10_(*q*‐value) for DEGs where up‐ and downregulations in tumor samples are shown in red and blue colors, respectively. The first and third columns were calculated using all DEGs (935 up‐ and 1387 downregulated DEGs), and the second and fourth columns were obtained by using subset of DEGs that were targets of DEmiRs (452 up‐ and 562 downregulated DEGs). The black and white heatmap on the right indicates presence or absence of miRNAs targeting DEGs involved for each process where validated and predicted targets are indicated in black and gray colors, respectively. Target genes with literature evidence are marked with an asterisk where further details were provided in Table S5.

Next, we characterized biological processes represented by the 2322 DEGs. Functional enrichment analysis for 935 up‐ and 1387 downregulated DEGs using 50 hallmarks gene sets of MSigDB revealed 24 hallmark signatures (Fig. 3). Representative activated processes included cell cycle regulators such as the G2M_checkpoint, E2F_targets, and the mitotic spindle. Most processes related to inflammation and immune signaling were downregulated. Epithelial–mesenchymal transition, estrogen responses, KRAS signaling, and hypoxia signaling showed mixed enrichments, suggesting their important but complicated regulatory roles.

### 3.3 Integrative analysis of miRNA and mRNA expression using miRNA–target information

Molecular and cellular functions of miRNAs are typically inferred through functions of their target mRNAs. However, accurate prediction of miRNA targets is challenging because miRNA targeting is mediated in a complicated manner by many functional site types and there are still unknown biological features that affect the efficacy of miRNA targeting (Kim *et al*., 2016). Having both miRNA‐Seq and mRNA‐Seq data from matched tumor–normal samples is a big advantage because it provides an opportunity to explore the negative correlation of miRNA and mRNA expressions, a condition that meaningful miRNA‐mRNA target pairs must satisfy.

We sought miRNA and target gene pairs (a) where both the miRNA and the target gene were differentially regulated, (b) whose directions of differential expression were opposite to each other, and (c) that were associated by miRNA targeting as compiled by merging contents in miRGator 3.0 and the latest TargetScan 7.0 predictions (Materials and methods section). We identified 1948 DEmiR‐DEG paired relations, covering 44 DEmiRs (22 and 22 up‐ and downregulated in tumor, respectively) and 1014 DEGs (452 and 562 up‐ and downregulated in tumor, respectively). Such DEmiR‐DEG pairs have a good chance to be functional in the pathophysiology of LUAD.

We performed gene set analysis for the 1014 target DEGs regulated by DEmiRs using the MSigDB hallmark gene sets (Fig. 3). Enriched biological processes for miRNA–target DEGs included the G2M_checkpoint and E2F_targets for activated pathways and TNFα_signaling_via_NFκB and Inflammatory_response for suppressed processes. DEmiRs that target genes in each enriched process were investigated using our compilation of miRNA–target information and the recently updated miRTarBase 7.0 knowledgebase (Fig. 3 and Table S5) (Chou *et al*., 2018). To identify biological processes under miRNA regulation, we looked for biological processes enriched by both DEGs and DEmiR targets. We identified several miRNAs known to target specific processes from literature survey (marked as an asterisk in Fig. 3). For example, miR‐138‐5p and miR‐144‐3p are known to target the EZH2 gene (Guo *et al*., 2013; Liang *et al*., 2013) and miR‐30a‐5p targets the MYBL gene to regulate the G2M_checkpoint and E2F_target processes (Martinez *et al*., 2011). However, many processes did not have literature basis to identify regulatory miRNAs and their targets, including the p53_pathway, glycolysis, estrogen_responses, KRAS_signaling, TNFa_signaling, inflammatory_response, and IL2_STAT5_signaling (Table S5). Thus, our analysis provides an ample opportunity for further functional studies.

### 3.4 Colony formation assay for DEmiRs identified 8 tumor suppressor miRNAs

Tumor suppressor miRNAs are of particular interest because they could potentially be developed into prognostic biomarkers or therapeutic RNA drugs since miRNA mimics can be used as anticancer agents (Adams *et al*., 2017). Colony formation assay was performed to screen for miRNAs with growth suppression ability. We subjectively selected 14 candidate miRNAs from the 26 downregulated DEmiRs from the ES_Korea data set based on the fold change ratio (logFC < −2), average expression level (logCPM > 10), and literature evidence (Table S6). Known TSmiRs were excluded from the candidates except miR‐486‐5p, which was used as a positive control.

**Figure 4:**
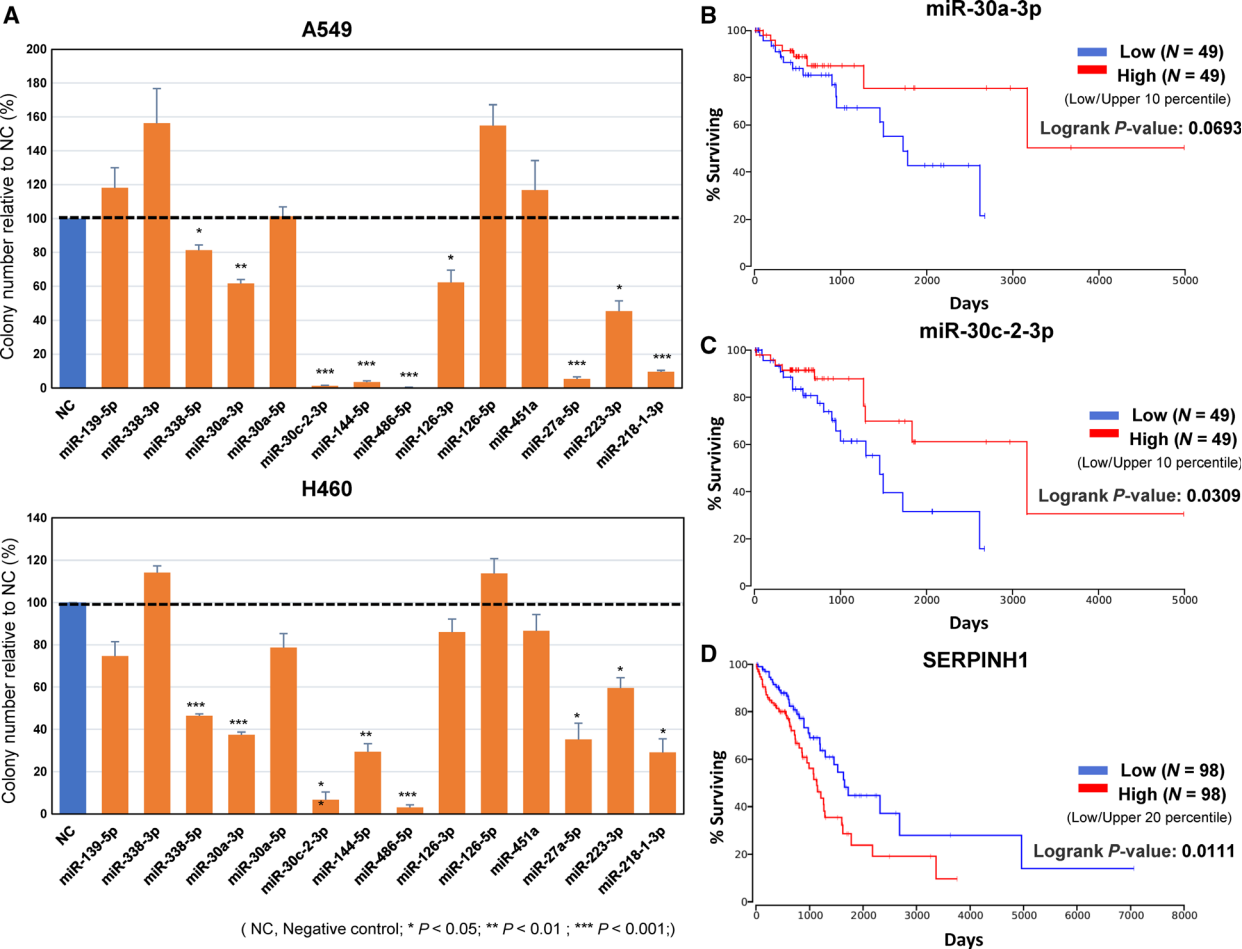
Functional and survival characteristics of TSmiR candidates. (A) Colony number relative to negative controls in the colony formation assay for A549 (top) and NCI‐H460 (bottom) cell lines. Error bars indicate the standard error of the mean. Each measurement was done in triplicate, and the *P*‐value was calculated with two‐tailed *t*‐test. (B‐D) Kaplan–Meier survival plots using the TCGA LUAD patients by expression value of miR‐30a‐3p (B), miR‐30c‐2‐3p (C), SERPINH1 (D), and the target gene of miR‐30c‐2‐3p.

Colony formation assays using A549 and NCI‐H460 human lung cancer cell lines revealed seven novel TSmiRs (miR‐338‐5p, miR‐30a‐5p, miR‐30c‐2‐3p, miR‐144‐5p, miR‐27a‐5p, miR‐223‐3p, miR‐218‐1‐3p), in addition to miR‐486‐5p used as the positive control, with tumor suppressor activity in both cell lines (Fig. 4A). High success rate, 8 out of 14 candidates, likely stemmed from the stringency in selecting DEmiRs from our data set.

Next, we investigated whether the expression level of the eight TSmiRs could serve as a prognostic marker for predicting patient survival. Using OncoLnc (http://www.oncolnc.org/) that provided online survival analysis for the TCGA patients (490 LUAD cases analyzed), we found that survival curves of two miRNAs, miR‐30a‐3p and miR‐30c‐2‐3p, showed meaningful separation between patient groups of top and bottom 10 percentiles in miRNA expression (Fig. 4B,C). Furthermore, SERPINH1, one of the predicted target genes of miR‐30c‐2‐3p, showed a clear separation between patient groups of top and bottom 20 percentiles (Fig. 4D). These data strongly suggest that miR‐30c‐2‐3p could be a TSmiR with prognostic value in LUAD.

### 3.5 Validation of candidate target genes for miRNAs with tumor suppressor activity

**Figure 5:**
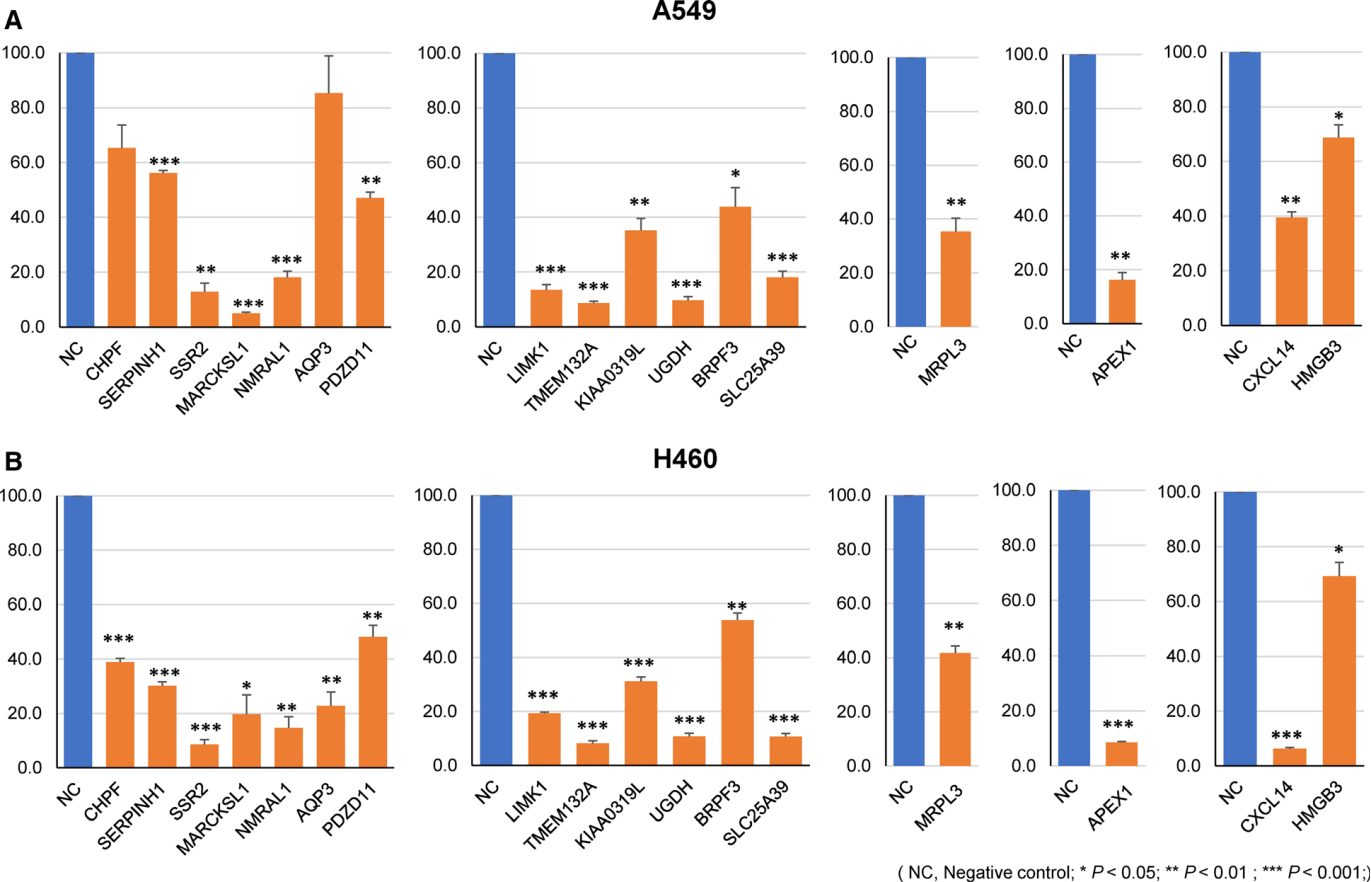
Relative change of target gene expression in qRT‐PCR experiment when cells were transfected with miRNA mimic in A549 (top) and NCI‐H460 (bottom) cell lines. Each measurement was done in triplicate and the *P*‐value was calculated with two‐tailed *t*‐test.

In an effort to identify target genes for TSmiRs whose activity was validated from the colony formation assay, we performed quantitative RT‐PCR to quantify mRNA expression changes resulting from transfection of a miRNA mimic of interest. For five miRNAs (miR‐30c‐2‐3p, miR‐144‐5p, miR‐486‐5p, miR‐27a‐5p, miR‐218‐1‐3p) that significantly reduced colony‐forming ability in two lung cancer cell lines, we selected 42 candidate target genes using a state‐of‐the‐art algorithm to predict miRNA–target genes (Materials and methods section) (Kim *et al*., 2016). We identified 17 target genes that showed reduced mRNA expression upon transfection with a miRNA mimic by more than 30% compared to the control sample in both cell lines (Fig. 5). Notably, SERPINH1 gene was one of the validated targets of miR‐30c‐2‐3p, again indicating its oncogenic function in agreement with the tumor‐suppressive role of miR‐30c‐2‐3p.

**Figure 6:**
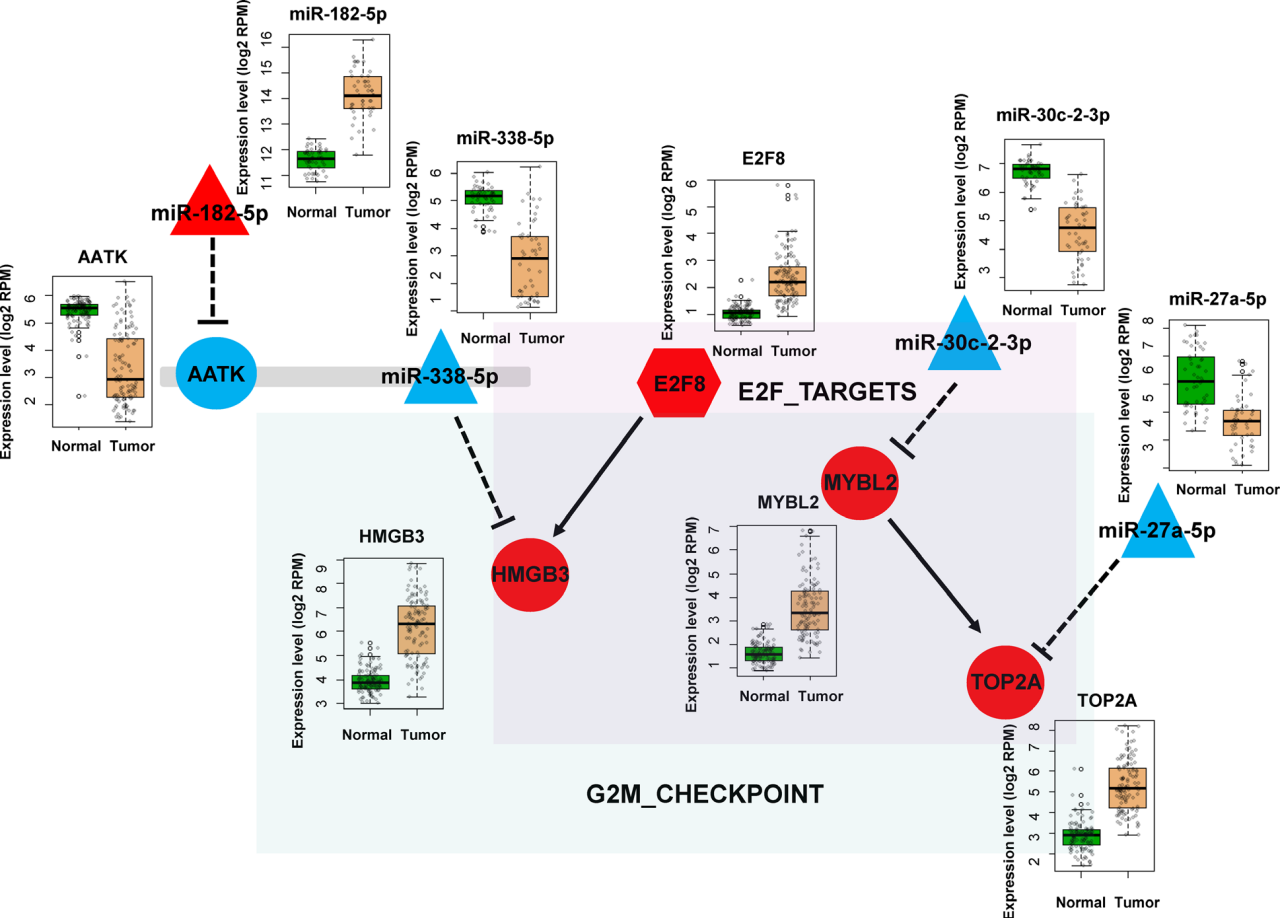
A network model of regulating cell cycles. All miRNAs and their target genes are differentially expressed between tumor and normal samples in concordant direction with negative regulation of miRNAs.

Several target genes (E2F8, MYBL2, HMGB3, and TOP2A) belonged to the gene sets of E2F_Targets and the G2M_Checkpoint, which were inferred from the MSigDB enrichment analysis. We constructed a network model for regulating the cell cycle by collecting miRNAs and their target genes, which were validated experimentally (Fig. 6). This network module is highly likely to be functional in tumorigenesis of lung cancer since all miRNA and genes were differentially regulated between tumor and normal tissues (see the Discussion section below).

## 4 Discussion

Studies that have sequenced mRNAs and miRNAs simultaneously are uncommon even though its benefit is well acknowledged. This is most likely due to the difficulties in generating miRNA‐Seq data. Experimental design of matched tumor–normal samples requires additional efforts in the sample acquisition process. Thus, our data set, providing both miRNA‐Seq and mRNA‐Seq data for matched tumor–normal samples from 48 Korean patients with LUAD, is a unique resource that includes ideal control data to avoid person‐to‐person variations when comparing tumor vs. normal or mRNA vs. miRNA expressions. This may serve as an optimal data set to test and evaluate advanced algorithms for identifying miRNA targets, discovering cancer biomarkers, or deciphering molecular mechanisms in tumorigenesis.

Our efforts were focused on identifying TSmiRs and target oncogenes that could be developed into prognostic biomarkers or therapeutic targets of cancer drugs. We identified seven novel TSmiRs with demonstrated tumor‐suppressive activity and 17 target genes whose expression was dependent on miRNA transfection in two lung cancer cell lines. Many of those TSmiRs and target genes were reported as prognostic markers in diverse types of tumor. Examples include miR‐30c‐2‐3p in breast cancer (Shukla *et al*., 2015), miR‐218 in hepatocellular carcinoma (Yang *et al*., 2016), CXCL14 in colorectal carcinoma (Zeng *et al*., 2013), and SERPINH1 in clear cell renal cell carcinoma (Qi *et al*., 2018).

Biological regulation is an elaborate process typically involving a complex network of regulatory loops. TP53 is a good example where more than 20 miRNAs are known to regulate TP53 by binding directly or targeting indirectly regulators of the p53 gene (Hermeking, 2012; Liu *et al*., 2017). On the other hand, TP53 itself regulates the transcriptional expression of a group of miRNAs, thus creating feedback loops.

We have discovered several DEmiRs and their target genes that are involved in cell cycle regulation (Fig. 6). E2F8 is a transcription factor for the HMGB3 gene whose overexpression is associated with poor prognosis in diverse types of cancer including non‐small cell lung cancer (Song *et al*., 2013). Notably, HMGB3 is the target of miR‐338‐5p that was consistently downregulated in LUAD (46 out of 48 patients). This miRNA is an intronic miRNA within the AATK gene that is also downregulated in tumor. Tumor‐suppressive roles of the hosting AATK gene are well established in melanoma and lung cancer cells (Haag *et al*., 2014; Ma and Rubin, 2014). We also observed that the AATK gene is the target of miR‐182‐5p that is upregulated in cancer. Thus, a regulatory cascade of miR‐182‐5p, the AATK gene, miR‐338‐5p and the E2F8 transcription factor possibly leads to overexpression of HMGB3.

Another regulatory network for the cell cycle consists of MYBL2 and TOP2A, which are targets of miR‐30c‐2‐3p and miR27a‐5p, respectively. Both genes are involved in the G2M_Checkpoint, and all members were differentially expressed in our data. MYBL2 is known to promote cell proliferation and EMT in many tumor types (Jin *et al*., 2017; Liang *et al*., 2017; Tao *et al*., 2015), and TOP2A is associated with worse prognosis in non‐small‐cell lung cancer patients (Hou *et al*., 2017).

Altogether, we present two regulatory paths responsible for cell cycle regulation. The detailed interplay of these regulatory elements remains to be elucidated, but our network model should enhance current understanding of the regulatory roles of miRNAs in LUAD.

## 5 Conclusions

Our study reports integrative analyses of high‐throughput sequencing data of miRNA and mRNA from 49 tumor–normal paired LUAD samples, which is the largest patient cohort of this kind for LUAD to date. We further identified and experimentally validated seven novel TSmiRs, two of which (miR‐30a‐3p and miR‐30c‐2‐3p) showing merits of prognostic molecular markers as well as potential targets for therapeutic manipulations. Multilayered deep sequencing with proper control samples followed by in‐depth integrative analysis proves to be a powerful approach to delineate molecular mechanisms behind cancer etiology and to identify molecular biomarkers of prognostic value.

## Conflict of interest

The authors declare no conflict of interest.

## Author contributions

SL, JK, and JK designed and supervised the research, and wrote the manuscript with contributions from all authors. HKK, JL, and YC managed the patient samples and clinical information. JEL and Seungjae L produced the deep sequencing data. NY, DYK, DB, YEL, and JS performed data organization, interpretation, and analyses. SY and YJ performed experiments.

## Supporting information


**Table S1.** Summary statistics of mapped reads and mapping rates.
**Table S2.** Sequence of oligonucleotide primers used in qRT‐PCR.
**Table S3.** Clinicopathological characteristics of lung adenocarcinoma patients.
**Table S4.** List of the 44 highly reliable DEmiRs including 18 up‐ and 26 downregulated miRNAs.
**Table S5.** List of miRNAs and target genes involved in the enriched biological processes.
**Table S6.** List of the 14 candidate miRNAs among the 26 downregulated DEmiRs from the ES_Korea data set based on the fold change ratio, average expression level, and literature evidences.
**Fig. S1.** In‐house workflows for analyzing miRNA‐Seq and RNA‐Seq data.
**Fig. S2.** Computational pipeline to identify differentially expressed miRNAs (DEmiRs) and genes (DEGs).
**Fig. S3.** Expression box plots for 18 miRNAs up‐regulated in tumor samples of the ES_Korea cohort.
