# Supplementary material for: Identification of tumor suppressor miRNAs by integrative miRNA and mRNA sequencing of matched tumor–normal samples in lung adenocarcinoma
Source: Mol Oncol. 2019 Apr 18;13(6):1356–68. doi: 10.1002/1878-0261.12478 (PMC6547618; doi:10.1002/1878-0261.12478)
Supplement: Supplementary file 1 — Table S1. Summary statistics of mapped reads and mapping rates. Table S2. Sequence of oligonucleotide primers used in qRT‐PCR. Table S3. Clinicopathological characteristics of lung adenocarcinoma patients. Table S4. List of the 44 highly reliable DEmiRs including 18 up‐ and 26 downregulated miRNAs. Table S5. List of miRNAs and target genes involved in the enriched biological processes. Table S6. List of the 14 candidate miRNAs among the 26 downregulated DEmiRs from the ES_Korea data set based on the fold change ratio, average expression level, and literature evidences. Fig. S1. In‐house workflows for analyzing miRNA‐Seq and RNA‐Seq data. Fig. S2. Computational pipeline to identify differentially expressed miRNAs (DEmiRs) and genes (DEGs). Fig. S3. Expression box plots for 18 miRNAs up‐regulated in tumor samples of the ES_Korea cohort. [file MOL2-13-1356-s001.pdf]

Supplementary Tables

Supplementary Table S1. Summary statistics of mapped reads and mapping rates

Supplementary Table S2. Sequence of oligonucleotide primers used in qRT-PCR

Supplementary Table S3. Clinicopathological characteristics of lung adenocarcinoma patients

Supplementary Table S4. List of the 44 highly reliable DEmiRs including 18 up- and 26 down-regulated miRNAs

Supplementary Table S5. List of miRNAs and target genes involved in the enriched biological processes. (a) miRNA-target gene relations with literature evidences for relevant functions. (b) miRNA-target gene relations with literature evidences for miRNA targeting. (c) miRNA-target gene relations based on prediction only.

Supplementary Table S6. List of the 14 candidate miRNAs among the 26 down-regulated DEmiRs from the ES_Korea data set based on the fold change ratio, average expression level, and literature evidences

Supplementary Figure Legends

Figure S1. In-house workflows for analyzing miRNA-Seq and RNA-Seq data.

Figure S2. Computational pipeline to identify differentially expressed miRNAs (DEmiRs) and genes (DEGs).

Figure S3. Expression box plots for 18 miRNAs up-regulated in tumor samples of the ES_Korea cohort.

Table S1. Summary statistics of mapped reads and mapping rates.

Sample	No. of raw reads	No. of reads mapped to			Percentage of reads mapped to		
		miRNA	ncRNA	genome	miRNA	ncRNA	genome
Pat01N	36,314,884	25,289,106	3,093,902	465,081	70.43%	8.62%	1.30%
Pat02N	34,279,617	24,879,755	1,380,218	487,422	76.15%	4.22%	1.49%
Pat05N	31,448,655	21,986,215	2,254,314	482,059	73.25%	7.51%	1.61%
Pat08N	17,939,023	10,954,432	2,694,042	342,080	61.71%	15.18%	1.93%
Pat15N	24,057,124	13,572,587	4,797,293	513,339	57.35%	20.27%	2.17%
Pat17N	43,042,497	29,989,098	2,727,941	485,653	70.96%	6.45%	1.15%
Pat27N	32,152,555	23,664,525	1,289,941	202,232	75.64%	4.12%	0.65%
Pat28N	34,340,900	25,734,195	1,655,740	433,614	75.99%	4.89%	1.28%
Pat29N	37,252,245	26,336,606	1,626,641	240,442	76.45%	4.72%	0.70%
Pat31N	30,453,647	20,817,125	1,799,126	301,225	70.47%	6.09%	1.02%
Pat32N	29,643,857	21,100,523	1,547,171	209,181	75.37%	5.53%	0.75%
Pat33N	34,971,831	20,954,418	5,075,403	447,681	61.41%	14.88%	1.31%
Pat36N	33,402,618	20,624,021	4,625,658	660,477	63.30%	14.20%	2.03%
Pat37N	20,361,977	13,123,127	2,352,104	343,148	67.70%	12.13%	1.77%
Pat38N	33,769,171	22,920,045	2,350,811	343,352	70.80%	7.26%	1.06%
Pat40N	31,962,476	22,198,653	2,779,080	367,698	70.62%	8.84%	1.17%
Pat42N	22,775,189	14,217,526	2,982,838	374,312	63.54%	13.33%	1.67%
Pat47N	37,130,389	24,435,562	3,530,352	620,023	67.63%	9.77%	1.72%
Pat49N	42,871,517	29,677,518	2,649,773	314,352	72.30%	6.46%	0.77%
Pat50N	20,761,542	13,408,809	2,658,844	333,180	65.43%	12.97%	1.63%
Pat56N	33,262,556	22,171,435	3,403,368	189,683	67.17%	10.31%	0.57%
Pat57N	32,220,262	21,142,155	3,223,178	413,490	68.21%	10.40%	1.33%
Pat58N	39,142,713	23,168,206	4,620,473	441,624	65.71%	13.10%	1.25%
Pat59N	27,425,794	14,960,591	4,314,489	456,972	60.34%	17.40%	1.84%
Pat60N	25,867,346	17,182,999	2,512,250	487,046	69.96%	10.23%	1.98%
Pat61N	16,441,331	9,050,822	3,379,969	199,775	57.49%	21.47%	1.27%
Pat64N	23,930,928	15,071,399	3,186,624	367,068	64.99%	13.74%	1.58%
Pat65N	20,488,848	12,350,864	3,631,992	209,229	62.30%	18.32%	1.06%
Pat66N	19,742,001	11,414,233	1,502,241	136,965	58.56%	7.71%	0.70%
Pat67N	26,759,242	16,069,720	1,324,074	328,181	63.89%	5.26%	1.30%
Pat68N	41,721,717	27,610,568	4,319,976	625,886	68.99%	10.79%	1.56%
Pat69N	32,530,707	17,683,243	4,295,229	349,691	55.09%	13.38%	1.09%
Pat75N	26,643,983	14,432,422	6,221,973	475,663	55.08%	23.74%	1.82%

Pat76N	43,675,341	22,820,738	10,009,047	866,699	53.15%	23.31%	2.02%
Pat78N	23,203,967	9,742,443	7,268,104	365,965	42.33%	31.58%	1.59%
Pat85N	25,976,039	15,358,907	4,655,031	424,640	59.62%	18.07%	1.65%
Pat88N	33,256,209	20,061,709	5,269,409	448,394	61.05%	16.04%	1.36%
Pat89N	25,835,379	15,988,914	3,648,329	267,485	62.66%	14.30%	1.05%
Pat95N	32,042,341	21,702,634	2,851,940	585,613	71.40%	9.38%	1.93%
Pat99N	12,086,579	6,258,719	3,394,954	152,585	52.72%	28.60%	1.29%
Pat102N	23,694,992	12,195,748	6,436,669	310,487	52.53%	27.72%	1.34%
Pat108N	28,791,005	20,561,882	1,728,932	308,516	73.20%	6.15%	1.10%
Pat109N	30,177,305	21,819,093	1,500,293	324,251	74.73%	5.14%	1.11%
Pat110N	42,852,133	25,398,244	7,781,898	708,984	60.61%	18.57%	1.69%
Pat111N	32,987,566	23,403,709	2,345,486	377,406	74.28%	7.44%	1.20%
Pat112N	28,667,538	18,451,387	3,492,437	420,890	66.14%	12.52%	1.51%
Pat113N	35,754,216	23,020,530	3,459,567	539,283	65.57%	9.85%	1.54%
Pat114N	37,599,503	25,229,953	1,829,165	272,057	73.73%	5.35%	0.80%
Pat01T	29,702,171	22,139,369	1,469,765	257,071	75.31%	5.00%	0.87%
Pat02T	29,980,260	22,787,803	1,124,025	293,762	77.97%	3.85%	1.01%
Pat05T	22,290,827	10,601,689	6,692,997	279,361	48.35%	30.53%	1.27%
Pat08T	19,400,973	10,789,132	4,106,843	187,865	56.23%	21.40%	0.98%
Pat15T	25,658,677	18,402,193	2,083,051	259,316	73.24%	8.29%	1.03%
Pat17T	34,623,659	25,362,937	1,585,925	253,754	74.31%	4.65%	0.74%
Pat27T	33,631,810	21,873,781	3,474,145	355,323	67.23%	10.68%	1.09%
Pat28T	36,803,337	23,729,396	4,313,887	643,513	66.84%	12.15%	1.81%
Pat29T	50,153,467	32,374,738	6,848,032	239,058	68.32%	14.45%	0.50%
Pat31T	35,921,283	23,001,342	4,015,488	425,739	66.65%	11.64%	1.23%
Pat32T	31,242,067	21,734,225	1,461,351	362,923	78.98%	5.31%	1.32%
Pat33T	29,248,399	16,773,410	5,540,171	388,328	59.24%	19.57%	1.37%
Pat36T	31,994,385	24,352,343	1,055,840	147,345	77.34%	3.35%	0.47%
Pat37T	33,689,492	21,988,494	4,900,494	426,686	65.95%	14.70%	1.28%
Pat38T	46,799,869	32,968,033	3,308,163	410,784	72.35%	7.26%	0.90%
Pat40T	35,628,420	22,587,246	5,546,884	304,357	63.99%	15.71%	0.86%
Pat42T	20,556,177	13,177,897	2,788,290	421,666	65.54%	13.87%	2.10%
Pat47T	33,407,210	23,323,591	3,028,428	653,893	72.62%	9.43%	2.04%
Pat49T	40,653,338	27,464,047	4,299,710	439,302	69.10%	10.82%	1.11%
Pat50T	35,174,672	22,772,467	5,498,781	369,872	65.75%	15.88%	1.07%
Pat56T	35,620,575	25,522,030	2,252,838	362,452	74.23%	6.55%	1.05%

Pat57T	33,432,177	23,637,156	1,600,462	280,929	74.84%	5.07%	0.89%
Pat58T	26,713,213	11,368,330	8,781,532	254,877	42.91%	33.14%	0.96%
Pat59T	26,475,665	12,428,918	7,103,726	282,865	50.06%	28.61%	1.14%
Pat60T	26,676,968	17,343,621	3,446,489	396,895	66.82%	13.28%	1.53%
Pat61T	21,689,194	11,914,570	5,148,631	268,173	55.98%	24.19%	1.26%
Pat64T	34,479,480	21,691,419	5,517,083	411,596	64.02%	16.28%	1.21%
Pat65T	29,841,258	16,965,785	6,840,822	228,029	57.42%	23.15%	0.77%
Pat66T	24,642,442	12,290,046	3,477,648	212,437	50.45%	14.27%	0.87%
Pat67T	24,454,364	11,367,013	4,656,489	210,696	47.84%	19.60%	0.89%
Pat68T	37,478,383	23,420,411	5,714,993	463,645	64.76%	15.80%	1.28%
Pat69T	31,285,095	14,593,418	6,601,538	403,520	47.25%	21.37%	1.31%
Pat75T	30,379,452	18,077,410	6,026,771	294,790	59.99%	20.00%	0.98%
Pat76T	26,305,050	12,660,716	8,023,297	312,909	48.47%	30.72%	1.20%
Pat78T	25,476,895	15,841,327	4,224,799	260,622	63.26%	16.87%	1.04%
Pat85T	32,146,260	20,087,678	5,079,661	430,284	62.95%	15.92%	1.35%
Pat88T	25,148,068	17,689,245	2,146,586	266,841	71.14%	8.63%	1.07%
Pat89T	24,197,838	12,844,224	6,134,235	313,341	53.67%	25.63%	1.31%
Pat95T	21,496,924	14,805,556	2,031,602	207,463	71.03%	9.75%	1.00%
Pat99T	24,995,829	13,976,383	5,471,555	226,868	56.72%	22.21%	0.92%
Pat102T	32,355,389	20,249,385	5,842,346	452,516	63.61%	18.35%	1.42%
Pat108T	37,169,641	26,098,031	3,765,948	379,964	72.03%	10.39%	1.05%
Pat109T	37,992,179	26,330,504	2,133,500	496,154	75.73%	6.14%	1.43%
Pat110T	34,038,089	24,053,359	2,697,961	302,789	73.60%	8.26%	0.93%
Pat111T	36,366,260	22,115,064	4,917,267	471,774	65.97%	14.67%	1.41%
Pat112T	42,488,554	16,943,134	13,174,519	1,543,636	41.76%	32.47%	3.80%
Pat113T	32,214,595	21,456,904	3,579,011	295,875	67.86%	11.32%	0.94%
Pat114T	36,437,529	26,065,821	1,516,436	361,211	76.67%	4.46%	1.06%

Table S2. Sequence of oligonucleotide primers used in qRT-PCR.

miR	Gene	Primer sequence (Forward)	Primer sequence (Reverse)
-	*ACTB*	acagagcctcgcctttgc	gaagccggccttgcacat
-	*HPRT1*	acactggcaaaacaatgcag	gtggggtccttttcaccag
miR-27a-5p	*APEX1*	tgccacactcaagatctgct	ctggggcttcttcctttacc
miR-144-5p	*MRPL3*	cgtccaggacagtatgtgga	tttcgtttgaccatgcgtag
miR-30c-2-3p	*AQP3*	agatgctccacatccgctac	cagccaaacatcaccaggat
	CHPF	ggtcgctgcattctcgat	gctcagctccagatggcta
	MARCKSL1	catcatgggcagccagag	tcacgtggccattctcct
	NMRAL1	tggtggacaagaaactggtg	cgaaccttgaatgtcccatc
	PDZD11	cagcatggattcctcctcat	tccaggaggcttcttcagtg
	SERPINH1	tgctagtcaacgccatgttc	cggtataggaccgagtcacc
	SSR2	ctgatgattccttccctcca	cacagtgtgggagacattgc
miR-218-1-3p	*BRPF3*	acggaatggtgtccctctta	gctcctccttcactgcactt
	KIAA0319L	catctgcgaagtggttgaga	aattgtgtcttcccctgctg
	LIMK1	agcgtggactttcagtctcc	cttcacatctgggctcatgc
	SLC25A39	cacgagcaccagcctagag	ctgaggccaccatttgct
	TMEM132A	cctggacgtcgtgagagtg	gaagtgttcaggggcgtcta
	UGDH	gatgggctccacaatgaact	tcggaatttcaccagaagga
miR-338-5p	*CXCL14*	gctacagcgacgtgaagaag	cgctcttggtggtgatgata
	HMGB3	cagctaagggaggcaagaag	gcggaattctgaacagaacag

Table S3. Clinicopathological characteristics of lung adenocarcinoma patients.

Num	Patient ID	Gender	Age	TNM Stage			Stage	Tumor type	Smoking	Chemotherapy	Radiotherapy
				p(T)	p(N)	p(M)					
1	Pat01	F	73	1b	0	0	1A	ADC	No	No	No
2	Pat02	F	57	2a	0	0	1B	ADC	No	No	No
3	Pat05	F	64	1b	0	0	1A	ADC	No	No	No
4	Pat08	F	45	2a	2	0	3A	ADC	No	No	No
5	Pat15	F	66	2a	0	0	1B	ADC	No	No	No
6	Pat17	F	73	2a	0	0	1B	ADC	No	No	No
7	Pat27	F	50	1b	0	0	1A	ADC	No	No	No
8	Pat28	F	78	1b	0	0	1A	ADC	No	No	No
9	Pat29	F	44	2a	2	0	3A	ADC	No	No	No
10	Pat31	F	54	1a	2	0	3A	ADC	No	No	No
11	Pat32	F	69	3	0	0	2B	ADC	No	No	No
12	Pat33	F	69	1a	0	0	1A	ADC	No	No	No
13	Pat36	F	37	1b	1	0	2A	ADC	No	No	No
14	Pat37	F	72	3	2	0	3A	ADC	No	No	No
15	Pat38	F	58	1a	0	0	1A	ADC	No	No	No
16	Pat40	F	70	2a	0	0	1B	ADC	No	No	No
17	Pat42	F	52	1a	0	0	1A	ADC	No	No	No
18	Pat47	F	56	1b	0	0	1A	ADC	No	No	No
19	Pat49	F	67	1a	0	0	1A	ADC	No	No	No
20	Pat50	F	56	1a	0	0	1A	ADC	No	No	No
21	Pat56	F	43	1b	1	0	2A	ADC	No	No	No
22	Pat57	F	52	1a	0	0	1A	ADC	No	No	No
23	Pat58	F	53	1a	0	0	1A	ADC	No	No	No
24	Pat59	F	74	1a	0	0	1A	ADC	No	No	No
25	Pat60	F	72	1a	0	0	1A	ADC	No	No	No
26	Pat61	F	67	1a	0	0	1A	ADC	No	No	No
27	Pat64	F	59	1a	1	0	2A	ADC	No	No	No
28	Pat65	F	70	2b	2	0	3A	ADC	No	No	No
29	Pat66	F	54	1a	0	0	1A	ADC	No	No	No
30	Pat67	F	56	1a	0	0	1A	ADC	No	No	No
31	Pat68	F	51	y2a	1	0	2A	ADC	No	Yes	No
32	Pat69	F	53	1b	2	0	3A	ADC	No	No	No
33	Pat75	F	48	1b	0	0	1A	ADC	No	No	No
34	Pat76	F	69	2a	0	0	1B	ADC	No	No	No
35	Pat78	F	46	2a	2	0	3A	ADC	No	No	No
36	Pat85	F	66	2a	1	0	2A	ADC	No	No	No
37	Pat88	F	50	2a	2	0	3A	ADC	No	No	No
38	Pat89	F	60	2a	2	0	3A	ADC	No	No	No
39	Pat95	F	66	2a	2	0	3A	ADC	No	No	No

40	Pat99	F	70	1a	0	0	1A	ADC	No	No	No
41	Pat102	F	57	1a	2	0	3A	ADC	No	No	No
42	Pat108	F	61	1a	2	0	3A	ADC	Yes	No	No
43	Pat109	F	61	1a	0	0	1A	ADC	Yes	No	No
44	Pat110	F	75	1a	0	0	1A	ADC	Yes	No	No
45	Pat111	F	66	2a	0	0	1B	ADC	Yes	No	No
46	Pat112	F	46	y1a	0	0	1A	ADC	Yes	YES	No
47	Pat113	F	62	2a	0	0	1B	ADC	Yes	No	No
48	Pat114	F	50	1a	0	0	1A	ADC	Yes	No	No

Table S4. List of the 44 highly reliable DEmiRs including 18 up- and 26 down-regulated miRNAs.

miRNAID		edgeR		DESeq2		Voom	
		log_2FC	AveExpr	log_2FC	AveExpr	log_2FC	AveExpr
Down-regulated miRNA	miR-1-3p	-1.06	3.59	-0.94	3.33	-1.16	3.15
	miR-126-3p	-1.95	12.85	-1.78	12.85	-1.96	12.40
	miR-126-5p	-1.89	14.51	-1.73	14.51	-1.90	14.14
	miR-133a-3p	-1.70	7.29	-1.27	7.28	-1.76	6.85
	miR-135a-5p	-2.32	3.76	-2.25	3.52	-2.29	3.04
	miR-138-5p	-2.12	6.29	-1.71	6.25	-2.14	5.68
	miR-139-5p	-2.34	6.21	-2.06	6.17	-2.39	5.62
	miR-144-3p	-2.31	9.54	-1.96	9.54	-2.30	8.65
	miR-144-5p	-2.46	9.16	-1.99	9.16	-2.42	8.40
	miR-145-3p	-1.43	8.56	-1.22	8.55	-1.47	8.16
	miR-204-5p	-1.93	6.46	-1.59	6.43	-1.92	5.96
	miR-218-5p	-1.92	8.22	-1.76	8.21	-1.93	7.85
	miR-218-1-3p	-1.86	5.82	-1.70	5.77	-1.91	5.38
	miR-223-3p	-1.26	8.92	-1.12	8.91	-1.26	8.60
	miR-223-5p	-1.31	4.18	-1.35	4.01	-1.31	3.67
	miR-27a-5p	-1.51	5.99	-1.58	5.94	-1.52	5.42
	miR-30a-3p	-2.45	9.89	-2.01	9.88	-2.46	9.25
	miR-30a-5p	-2.11	15.24	-1.82	15.24	-2.13	14.77
	miR-30c-2-3p	-2.05	6.14	-1.84	6.10	-2.09	5.65
	miR-338-3p	-2.78	9.41	-1.72	9.40	-2.77	8.56
	miR-338-5p	-2.23	4.64	-1.67	4.52	-2.26	3.95
	miR-451a	-2.25	12.99	-1.96	12.99	-2.24	12.31
	miR-486-3p	-1.54	3.98	-1.51	3.79	-1.52	3.41
	miR-486-5p	-2.49	13.80	-1.96	13.81	-2.47	13.01
	miR-511-5p	-1.07	4.52	-0.88	4.39	-1.14	4.15
	miR-584-5p	-1.62	4.15	-1.49	3.98	-1.62	3.68
Up-regulated miRNA	miR-130b-5p	1.32	4.64	1.37	4.52	1.27	4.33
	miR-135b-5p	2.74	6.45	2.92	6.42	2.69	5.62
	miR-148a-3p	1.28	14.82	1.43	14.83	1.28	14.53
	miR-182-5p	2.58	13.63	2.77	13.64	2.59	12.88
	miR-183-5p	2.66	9.95	2.95	9.95	2.66	9.08
	miR-200a-5p	1.60	5.30	1.66	5.22	1.58	4.91
	miR-200b-3p	1.63	10.33	1.81	10.33	1.64	9.92
	miR-200b-5p	1.34	4.10	1.43	3.92	1.28	3.71
	miR-21-3p	2.36	11.56	2.44	11.56	2.35	10.90
	miR-21-5p	2.33	16.04	2.25	16.04	2.32	15.54
	miR-210-3p	1.61	8.09	2.06	8.08	1.59	7.45
	miR-301b-3p	2.07	5.94	2.29	5.89	2.05	5.31
	miR-375	1.71	12.35	2.13	12.36	1.74	11.69
	miR-429	1.48	9.63	1.64	9.63	1.48	9.26
	miR-450b-5p	1.31	6.38	1.66	6.35	1.29	5.88
	miR-577	2.03	4.52	2.65	4.39	1.91	3.49
	miR-9-5p	2.24	5.21	2.80	5.13	2.17	4.15
	miR-96-5p	1.93	6.12	2.10	6.08	1.91	5.47

Table S5. List of miRNAs and target genes involved in the enriched biological processes.

(a) miRNA-target gene relations with literature evidences for relevant functions.

miR ID	Target gene symbol	MsigDB hallmark gene sets of target gene	Reference for relevant function
hsa-miR-133a-3p	COL1A1	EPITHELIAL_MESENCHYMAL_TRANSITION	Muraoka N, et al. MiR-133 promotes cardiac reprogramming by directly repressing Snai1 and silencing fibroblast signatures. EMBO J. 2014 Jul 17;33(14):1565-81.
hsa-miR-138-5p	EZH2	G2M_CHECKPOINT	Wang W, et al. MiR-138 induces cell cycle arrest by targeting cyclin D3 in hepatocellular carcinoma. Carcinogenesis. 2012 May;33(5):1113-20.
hsa-miR-138-5p	EZH2	E2F_TARGETS	Wang W, et al. MiR-138 induces cell cycle arrest by targeting cyclin D3 in hepatocellular carcinoma. Carcinogenesis. 2012 May;33(5):1113-20.
hsa-miR-139-5p	TOP2A	E2F_TARGETS	Huang LL, et al. Potential role of miR-139-5p in cancer diagnosis, prognosis and therapy. Oncol Lett. 2017 Aug;14(2):1215-1222.
hsa-miR-144-3p	EZH2	G2M_CHECKPOINT	Wu M, et al. MicroRNA-144-3p suppresses tumor growth and angiogenesis by targeting SGK3 in hepatocellular carcinoma. Oncol Rep. 2017 Oct;38(4):2173-2181.
hsa-miR-144-3p	EZH2	E2F_TARGETS	Wu M, et al. MicroRNA-144-3p suppresses tumor growth and angiogenesis by targeting SGK3 in hepatocellular carcinoma. Oncol Rep. 2017 Oct;38(4):2173-2181.
hsa-miR-148a-3p	TGFB2	APOPTOSIS	Baltruskeviciene E, et al. Down-regulation of miRNA-148a and miRNA-625-3p in colorectal cancer is associated with tumor budding. BMC Cancer. 2017 Sep 1;17(1):607.
hsa-miR-182-5p	BDNF	EPITHELIAL_MESENCHYMAL_TRANSITION	Shi J. Regulatory networks between neurotrophins and miRNAs in brain diseases and cancers. Acta Pharmacol Sin. 2015 Feb;36(2):149-57.
hsa-miR-182-5p	SNAI2	EPITHELIAL_MESENCHYMAL_TRANSITION	Qu Y, Li WC, et al. MiR-182 and miR-203 induce mesenchymal to epithelial transition and self-sufficiency of growth signals via repressing SNAI2 in prostate cells. Int J Cancer. 2013 Aug 1;133(3):544-55.
hsa-miR-21-5p	BTG2	APOPTOSIS	Liu M, et al. Regulation of the cell cycle gene, BTG, by miR-21 in human laryngeal carcinoma. Cell Res. 2009 Jul;19(7):828-37.
hsa-miR-21-5p	MYC	ESTROGEN_RESPONSE_EARLY	Bhat-Nakshatri P, et al. Estradiol-regulated microRNAs control estradiol response in breast cancer cells. Nucleic Acids Res. 2009 Aug;37(14):4850-61.
hsa-miR-21-5p	RHOB	EPITHELIAL_MESENCHYMAL_TRANSITION	Ding XM. MicroRNAs: regulators of cancer metastasis and epithelial-mesenchymal transition (EMT). Chin J Cancer. 2014 Mar;33(3):140-7.
hsa-miR-21-5p	TGFBR3	APOPTOSIS	Tonevitsky AG, et al. Dynamically regulated miRNA-mRNA networks revealed by exercise. BMC Physiol. 2013 Jun 7;13:9.
hsa-miR-21-5p	TIMP3	EPITHELIAL_MESENCHYMAL_TRANSITION	Martin del Campo SE, et al. MiR-21 enhances melanoma invasiveness via inhibition of tissue inhibitor of metalloproteinases 3 expression: in vivo effects of MiR-21 inhibitor. PLoS One. 2015 Jan 14;10(1):e0115919
hsa-miR-21-5p	TIMP3	APOPTOSIS	Martin del Campo SE, et al. MiR-21 enhances melanoma invasiveness via inhibition of tissue inhibitor of metalloproteinases 3 expression: in vivo effects of MiR-21 inhibitor. PLoS One. 2015 Jan 14;10(1):e0115919
hsa-miR-30a-5p	MYBL	G2M_CHECKPOINT	Martinez I, et al. miR-29 and miR-30 regulate B-Myb expression during cellular senescence. Proc Natl Acad Sci U S A. 2011 Jan 11;108(2):522-7.
hsa-miR-30a-5p	MYBL	E2F_TARGETS	Martinez I, et al. miR-29 and miR-30 regulate B-Myb expression during cellular senescence. Proc Natl Acad Sci U S A. 2011 Jan 11;108(2):522-7.

hsa-miR-30a-5p	NT5E	EPITHELIAL_MESENCHYMAL_TRANSITION	Zhu J, et al. CD73/NT5E is a target of miR-30a-5p and plays an important role in the pathogenesis of non-small cell lung cancer. Mol Cancer. 2017 Feb 3;16(1):34.
hsa-miR-375	CTGF	EPITHELIAL_MESENCHYMAL_TRANSITION	Kang W, et al. miR-375 is involved in Hippo pathway by targeting YAP1/TEAD4-CTGF axis in gastric carcinogenesis. Cell Death Dis. 2018 Jan 24;9(2):92.
hsa-miR-375	CTGF	HYPOXIA	Ou J, et al. MiR-375 attenuates injury of cerebral ischemia/reperfusion via targetting Ctgf. Biosci Rep. 2017 Dec 22;37(6). pii: BSR20171242.
hsa-miR-96-5p	SLC1A1	ESTROGEN_RESPONSE_EARLY	Dambal S, et al. The microRNA-183 cluster: the family that plays together stays together. Nucleic Acids Res. 2015 Sep 3;43(15):7173-88.

(b) miRNA-target gene relations with literature evidences for miRNA targeting.

miR ID	Target gene symbol	PubMed ID for target information	MsigDB hallmark gene sets of target gene
hsa-miR-126-3p	SLC7A5	21439283, 20371350, 26054677, 26244545	ESTROGEN_RESPONSE_EARLY, ESTROGEN_RESPONSE_LATE, G2M_CHECKPOINT
hsa-miR-126-3p	VCAM1	18227515	EPITHELIAL_MESENCHYMAL_TRANSITION
hsa-miR-133a-3p	COL1A1	21769867	EPITHELIAL_MESENCHYMAL_TRANSITION
hsa-miR-133a-3p	MMP14	23783274	EPITHELIAL_MESENCHYMAL_TRANSITION
hsa-miR-135a-5p	MMP11	27323416	KRAS_SIGNALING_UP
hsa-miR-135b-5p	KLF4	20981674, 26877610	TNFA_SIGNALING_VIA_NFKB, KRAS_SIGNALING_UP, ESTROGEN_RESPONSE_EARLY, ESTROGEN_RESPONSE_LATE
hsa-miR-138-5p	EZH2	23343715, 21770894, 23707559, 25339353, 27019355, 27266699	E2F_TARGETS, G2M_CHECKPOINT
hsa-miR-138-5p	HMGA1	22012620	E2F_TARGETS, G2M_CHECKPOINT
hsa-miR-138-5p	SOX9	27347323	GLYCOLYSIS, KRAS_SIGNALING_UP
hsa-miR-1-3p	ADAM12	18668037	EPITHELIAL_MESENCHYMAL_TRANSITION
hsa-miR-1-3p	MET	19710019, 18818206, 18593903, 18668040, 21169019, 18668037, 25874496, 27247259	GLYCOLYSIS
hsa-miR-1-3p	SOX9	23352489	GLYCOLYSIS, KRAS_SIGNALING_UP
hsa-miR-144-3p	EZH2	23815091	E2F_TARGETS, G2M_CHECKPOINT
hsa-miR-148a-3p	DNAJB4	20371350	TNFA_SIGNALING_VIA_NFKB
hsa-miR-148a-3p	GAS1	20371350, 23446348, 21572407	EPITHELIAL_MESENCHYMAL_TRANSITION
hsa-miR-148a-3p	ITGA5	21703006	EPITHELIAL_MESENCHYMAL_TRANSITION, INFLAMMATORY_RESPONSE
hsa-miR-148a-3p	KLF6	23592263	TNFA_SIGNALING_VIA_NFKB, INFLAMMATORY_RESPONSE, IL2_STAT5_SIGNALING, HYPOXIA
hsa-miR-148a-3p	LDLR	27292025	TNFA_SIGNALING_VIA_NFKB, INFLAMMATORY_RESPONSE
hsa-miR-148a-3p	SIK1	23592263, 23446348, 21572407	TNFA_SIGNALING_VIA_NFKB
hsa-miR-148a-3p	SLC2A3	23622248	TNFA_SIGNALING_VIA_NFKB, IL2_STAT5_SIGNALING, HYPOXIA
hsa-miR-148a-3p	TGFB2	26983401	APOPTOSIS
hsa-miR-182-5p	BDNF	23704927, 25955435	EPITHELIAL_MESENCHYMAL_TRANSITION
hsa-miR-182-5p	CADM1	24445397	EPITHELIAL_MESENCHYMAL_TRANSITION
hsa-miR-182-5p	SNAI2	23354685	EPITHELIAL_MESENCHYMAL_TRANSITION
hsa-miR-183-5p	AKAP12	20979053	KRAS_SIGNALING_UP, HYPOXIA

hsa-miR-183-5p	EGR1	21118966	TNFA_SIGNALING_VIA_NFKB
hsa-miR-200b-3p	DLC1	23708087	ESTROGEN_RESPONSE_EARLY
hsa-miR-200b-3p	FERMT2	24064224	EPITHELIAL_MESENCHYMAL_TRANSITION
hsa-miR-204-5p	MMP9	28280358	KRAS_SIGNALING_UP
hsa-miR-210-3p	BDNF	19826008, 26708520	EPITHELIAL_MESENCHYMAL_TRANSITION
hsa-miR-21-5p	BTG2	19546886, 20371350, 18591254, 23857284, 24821435, 23446999	TNFA_SIGNALING_VIA_NFKB, INFLAMMATORY_RESPONSE, APOPTOSIS
hsa-miR-21-5p	CADM1	27055844	EPITHELIAL_MESENCHYMAL_TRANSITION
hsa-miR-21-5p	IL1B	21131358	TNFA_SIGNALING_VIA_NFKB, INFLAMMATORY_RESPONSE, KRAS_SIGNALING_UP, APOPTOSIS
hsa-miR-21-5p	MYC	19528081, 24510096	TNFA_SIGNALING_VIA_NFKB, INFLAMMATORY_RESPONSE, IL2_STAT5_SIGNALING, ESTROGEN_RESPONSE_EARLY
hsa-miR-21-5p	OLR1	18591254	TNFA_SIGNALING_VIA_NFKB, INFLAMMATORY_RESPONSE
hsa-miR-21-5p	RHOB	20460403, 21347332, 21872591, 23313253	TNFA_SIGNALING_VIA_NFKB, EPITHELIAL_MESENCHYMAL_TRANSITION, IL2_STAT5_SIGNALING, APOPTOSIS
hsa-miR-21-5p	TGFBR3	18829576, 19253296	EPITHELIAL_MESENCHYMAL_TRANSITION, APOPTOSIS
hsa-miR-21-5p	TIMP3	18591254, 20346171, 21820586, 23504349, 24574341	EPITHELIAL_MESENCHYMAL_TRANSITION, APOPTOSIS
hsa-miR-21-5p	WFS1	19253296	ESTROGEN_RESPONSE_EARLY, ESTROGEN_RESPONSE_LATE
hsa-miR-218-5p	BIRC5	21385904, 23212916, 23446348, 21572407, 20371350, 26442524, 25900794	E2F_TARGETS, G2M_CHECKPOINT, MITOTIC_SPINDLE
hsa-miR-218-5p	LMNB1	20371350	E2F_TARGETS, G2M_CHECKPOINT, MITOTIC_SPINDLE
hsa-miR-27a-5p	GREM1	27910957	EPITHELIAL_MESENCHYMAL_TRANSITION
hsa-miR-301b-3p	DLC1	20371350	ESTROGEN_RESPONSE_EARLY
hsa-miR-301b-3p	EDN1	23592263	TNFA_SIGNALING_VIA_NFKB, INFLAMMATORY_RESPONSE
hsa-miR-301b-3p	KLF6	23592263	TNFA_SIGNALING_VIA_NFKB, INFLAMMATORY_RESPONSE, IL2_STAT5_SIGNALING, HYPOXIA
hsa-miR-301b-3p	LDLR	22012620, 21572407, 20371350, 27292025	TNFA_SIGNALING_VIA_NFKB, INFLAMMATORY_RESPONSE
hsa-miR-301b-3p	SIK1	23592263, 23446348, 21572407	TNFA_SIGNALING_VIA_NFKB
hsa-miR-30a-5p	FUCA1	22473208	EPITHELIAL_MESENCHYMAL_TRANSITION, KRAS_SIGNALING_UP, P53_PATHWAY
hsa-miR-30a-5p	ITGA2	18668040	EPITHELIAL_MESENCHYMAL_TRANSITION, KRAS_SIGNALING_UP
hsa-miR-30a-5p	KIF11	18668040, 21572407, 20371350	G2M_CHECKPOINT, MITOTIC_SPINDLE
hsa-miR-30a-5p	MET	18668040	GLYCOLYSIS
hsa-miR-30a-5p	MYBL2	22473208	E2F_TARGETS, G2M_CHECKPOINT
hsa-miR-30a-5p	NT5E	18668040, 28158983	EPITHELIAL_MESENCHYMAL_TRANSITION, GLYCOLYSIS
hsa-miR-30a-5p	SLC7A11	18668040	P53_PATHWAY
hsa-miR-30a-5p	SLC7A5	23592263	ESTROGEN_RESPONSE_EARLY, ESTROGEN_RESPONSE_LATE, G2M_CHECKPOINT
hsa-miR-30c-2-3p	HMGA1	26701625	E2F_TARGETS, G2M_CHECKPOINT
hsa-miR-375	CTGF	20215506	EPITHELIAL_MESENCHYMAL_TRANSITION, HYPOXIA
hsa-miR-375	KLF4	27279635	TNFA_SIGNALING_VIA_NFKB, KRAS_SIGNALING_UP, ESTROGEN_RESPONSE_EARLY, ESTROGEN_RESPONSE_LATE
hsa-miR-429	DLC1	27602157	ESTROGEN_RESPONSE_EARLY
hsa-miR-450b-5p	REEP1	27418678	ESTROGEN_RESPONSE_EARLY, MYOGENESIS
hsa-miR-451a	MMP9	20816946	KRAS_SIGNALING_UP
hsa-miR-486-3p	HMGA1	26701625	E2F_TARGETS, G2M_CHECKPOINT
hsa-miR-486-3p	KRT8	24398324	ESTROGEN_RESPONSE_EARLY

hsa-miR-486-3p	SFN	26701625	ESTROGEN_RESPONSE_EARLY, ESTROGEN_RESPONSE_LATE, P53_PATHWAY
hsa-miR-486-5p	CIT	26183718	E2F_TARGETS
hsa-miR-96-5p	SLC1A1	24304186	ESTROGEN_RESPONSE_EARLY

(c) miRNA-target gene relations based on prediction only.

miR ID	Target gene symbol	Prediction programs	MsigDB hallmark gene sets of target gene
hsa-miR-130b-5p	ABLIM1	TargetScan	ESTROGEN_RESPONSE_EARLY, MYOGENESIS
hsa-miR-130b-5p	CXCL2	TargetScan, MicrocosmTargets	TNFA_SIGNALING_VIA_NFKB
hsa-miR-130b-5p	DST	TargetScan	EPITHELIAL_MESENCHYMAL_TRANSITION
hsa-miR-130b-5p	IGFBP6	TargetScan, MicrocosmTargets	APOPTOSIS
hsa-miR-130b-5p	IL33	TargetScan, miRDB	KRAS_SIGNALING_UP
hsa-miR-130b-5p	IL7R	TargetScan, MicrocosmTargets, miRDB	TNFA_SIGNALING_VIA_NFKB, INFLAMMATORY_RESPONSE, KRAS_SIGNALING_UP
hsa-miR-130b-5p	PCOLCE2	TargetScan	EPITHELIAL_MESENCHYMAL_TRANSITION
hsa-miR-130b-5p	PTGER3	TargetScan, miRDB	ESTROGEN_RESPONSE_LATE
hsa-miR-130b-5p	SLC1A1	TargetScan, miRDB	ESTROGEN_RESPONSE_EARLY
hsa-miR-130b-5p	TNFSF1	TargetScan	INFLAMMATORY_RESPONSE, IL2_STAT5_SIGNALING, APOPTOSIS
hsa-miR-133a-3p	ADAM12	TargetScan	EPITHELIAL_MESENCHYMAL_TRANSITION
hsa-miR-133a-3p	COL6A3	TargetScan	EPITHELIAL_MESENCHYMAL_TRANSITION
hsa-miR-133a-3p	ELF3	TargetScan	ESTROGEN_RESPONSE_EARLY, GLYCOLYSIS
hsa-miR-133a-3p	NME1	TargetScan	E2F_TARGETS
hsa-miR-135a-5p	ADAM12	TargetScan, miRNAorg, PITA	EPITHELIAL_MESENCHYMAL_TRANSITION
hsa-miR-135a-5p	COL5A1	TargetScan, miRNAorg, PITA	EPITHELIAL_MESENCHYMAL_TRANSITION, GLYCOLYSIS
hsa-miR-135a-5p	DEPTOR	miRNAorg, MicrocosmTargets, PITA	ESTROGEN_RESPONSE_EARLY
hsa-miR-135a-5p	FRK	TargetScan, miRNAorg, MicrocosmTargets, PITA, PicTar, miRDB	ESTROGEN_RESPONSE_EARLY, ESTROGEN_RESPONSE_LATE
hsa-miR-135a-5p	HKDC1	miRNAorg, MicrocosmTargets, PITA	KRAS_SIGNALING_UP
hsa-miR-135a-5p	ITGA2	miRNAorg, PITA, miRDB	EPITHELIAL_MESENCHYMAL_TRANSITION, KRAS_SIGNALING_UP
hsa-miR-135a-5p	MXRA5	miRNAorg, MicrocosmTargets, PITA, miRDB	EPITHELIAL_MESENCHYMAL_TRANSITION
hsa-miR-135a-5p	SFRP4	TargetScan, miRNAorg, PITA	EPITHELIAL_MESENCHYMAL_TRANSITION
hsa-miR-135a-5p	THBS2	miRNAorg, PITA, miRDB	EPITHELIAL_MESENCHYMAL_TRANSITION
hsa-miR-135a-5p	VCAN	TargetScan, miRNAorg, PITA, miRDB	EPITHELIAL_MESENCHYMAL_TRANSITION, GLYCOLYSIS
hsa-miR-135b-5p	ATF3	TargetScan, miRNAorg, PicTar	TNFA_SIGNALING_VIA_NFKB, HYPOXIA, APOPTOSIS
hsa-miR-135b-5p	CXCL12	TargetScan, miRNAorg, PITA, PicTar	EPITHELIAL_MESENCHYMAL_TRANSITION, ESTROGEN_RESPONSE_EARLY, ESTROGEN_RESPONSE_LATE
hsa-miR-135b-5p	EMP1	TargetScan, miRNAorg, PITA	IL2_STAT5_SIGNALING, KRAS_SIGNALING_UP, APOPTOSIS
hsa-miR-135b-5p	FERMT2	TargetScan, miRNAorg, PITA	EPITHELIAL_MESENCHYMAL_TRANSITION
hsa-miR-135b-5p	HBEGF	miRNAorg, MicrocosmTargets, PITA	TNFA_SIGNALING_VIA_NFKB, INFLAMMATORY_RESPONSE, KRAS_SIGNALING_UP, MYOGENESIS
hsa-miR-135b-5p	PLAGL1	TargetScan, miRNAorg, MicrocosmTargets, PITA, miRDB	IL2_STAT5_SIGNALING

hsa-miR-135b-5p	VLDLR	TargetScan, miRNAorg, MicrocosmTargets, PITA, PicTar, miRDB	HYPOXIA
hsa-miR-138-5p	INHBB	TargetScan, PITA, PicTar	ESTROGEN_RESPONSE_EARLY, P53_PATHWAY
hsa-miR-138-5p	MDK	miRNAorg, MicrocosmTargets, PITA	ESTROGEN_RESPONSE_LATE
hsa-miR-138-5p	PPFIA4	TargetScan, miRNAorg, PITA	GLYCOLYSIS
hsa-miR-138-5p	UBE2C	miRNAorg, MicrocosmTargets, PITA	G2M_CHECKPOINT
hsa-miR-139-5p	ADAM12	miRNAorg, PITA, miRDB	EPITHELIAL_MESENCHYMAL_TRANSITION
hsa-miR-139-5p	CDCA8	miRNAorg, MicrocosmTargets, PITA	E2F_TARGETS
hsa-miR-139-5p	EZH2	miRNAorg, MicrocosmTargets, PITA	E2F_TARGETS, G2M_CHECKPOINT
hsa-miR-139-5p	GALNT3	TargetScan, miRNAorg, PITA, miRDB	KRAS_SIGNALING_UP
hsa-miR-139-5p	TOP2A	miRNAorg, PITA, miRDB	E2F_TARGETS, ESTROGEN_RESPONSE_LATE, G2M_CHECKPOINT, MITOTIC_SPINDLE
hsa-miR-144-3p	COL11A1	miRNAorg, MicrocosmTargets, PITA	EPITHELIAL_MESENCHYMAL_TRANSITION
hsa-miR-144-3p	COL5A2	TargetScan, miRNAorg, PITA	EPITHELIAL_MESENCHYMAL_TRANSITION
hsa-miR-144-3p	DNAJC12	miRNAorg, MicrocosmTargets, PITA, miRDB	ESTROGEN_RESPONSE_LATE
hsa-miR-144-3p	E2F8	miRNAorg, MicrocosmTargets, PITA, PicTar, miRDB	E2F_TARGETS
hsa-miR-144-3p	ERO1L	TargetScan, miRNAorg	GLYCOLYSIS, KRAS_SIGNALING_UP
hsa-miR-144-3p	GALNT3	miRNAorg, PITA, miRDB	KRAS_SIGNALING_UP
hsa-miR-144-3p	HS6ST2	miRNAorg, MicrocosmTargets, PITA	GLYCOLYSIS
hsa-miR-144-3p	MXRA5	TargetScan, miRNAorg, PITA, PicTar, miRDB	EPITHELIAL_MESENCHYMAL_TRANSITION
hsa-miR-144-3p	PMAIP1	TargetScan, miRNAorg, PITA	ESTROGEN_RESPONSE_EARLY
hsa-miR-144-3p	SLC12A2	TargetScan, miRNAorg, PITA, PicTar, miRDB	G2M_CHECKPOINT
hsa-miR-144-3p	SLC7A11	TargetScan, miRNAorg, PITA, PicTar, miRDB	P53_PATHWAY
hsa-miR-144-3p	STIL	TargetScan, miRNAorg, miRDB	ESTROGEN_RESPONSE_LATE, G2M_CHECKPOINT
hsa-miR-144-3p	TNC	TargetScan, miRNAorg	EPITHELIAL_MESENCHYMAL_TRANSITION
hsa-miR-144-3p	TOP2A	TargetScan, miRNAorg, PITA, miRDB	E2F_TARGETS, ESTROGEN_RESPONSE_LATE, G2M_CHECKPOINT, MITOTIC_SPINDLE
hsa-miR-144-5p	GPX2	TargetScan	P53_PATHWAY
hsa-miR-145-3p	CDK1	TargetScan	E2F_TARGETS, G2M_CHECKPOINT, GLYCOLYSIS, MITOTIC_SPINDLE
hsa-miR-145-3p	HS6ST2	TargetScan, miRDB	GLYCOLYSIS
hsa-miR-145-3p	ITGBL1	TargetScan	KRAS_SIGNALING_UP
hsa-miR-148a-3p	APLNR	miRNAorg, MicrocosmTargets, PITA	INFLAMMATORY_RESPONSE, MYOGENESIS
hsa-miR-148a-3p	CADM1	TargetScan, miRNAorg, PITA	EPITHELIAL_MESENCHYMAL_TRANSITION
hsa-miR-148a-3p	DUSP1	miRNAorg, PITA, PicTar	TNFA_SIGNALING_VIA_NFKB, HYPOXIA
hsa-miR-148a-3p	EGR3	TargetScan, miRNAorg, PITA	TNFA_SIGNALING_VIA_NFKB, APOPTOSIS, ESTROGEN_RESPONSE_EARLY, ESTROGEN_RESPONSE_LATE
hsa-miR-148a-3p	EMP1	miRNAorg, PITA, miRDB	IL2_STAT5_SIGNALING, KRAS_SIGNALING_UP, APOPTOSIS
hsa-miR-148a-3p	F3	TargetScan, miRNAorg, MicrocosmTargets, PITA	TNFA_SIGNALING_VIA_NFKB, INFLAMMATORY_RESPONSE, HYPOXIA
hsa-miR-148a-3p	FOSB	TargetScan, miRNAorg, PITA, PicTar	TNFA_SIGNALING_VIA_NFKB
hsa-miR-148a-3p	GLIPR2	miRNAorg, MicrocosmTargets, PITA	IL2_STAT5_SIGNALING
hsa-miR-148a-3p	IL1RL1	miRNAorg, MicrocosmTargets, PITA	IL2_STAT5_SIGNALING
hsa-miR-148a-3p	KLF4	TargetScan, miRNAorg, MicrocosmTargets, PITA, PicTar	TNFA_SIGNALING_VIA_NFKB, KRAS_SIGNALING_UP, ESTROGEN_RESPONSE_EARLY, ESTROGEN_RESPONSE_LATE
hsa-miR-148a-3p	LDB3	miRNAorg, PITA, miRDB	MYOGENESIS

hsa-miR-148a-3p	MAFF	miRNAorg, PITA, miRDB	TNFA_SIGNALING_VIA_NFKB, IL2_STAT5_SIGNALING, HYPOXIA
hsa-miR-148a-3p	MGP	miRNAorg, MicrocosmTargets, PITA	EPITHELIAL_MESENCHYMAL_TRANSITION
hsa-miR-148a-3p	NDST1	miRNAorg, PITA, PicTar	HYPOXIA
hsa-miR-148a-3p	PAPSS2	miRNAorg, MicrocosmTargets, PITA, miRDB	ESTROGEN_RESPONSE_EARLY, ESTROGEN_RESPONSE_LATE
hsa-miR-148a-3p	PPAP2B	TargetScan, PITA	TNFA_SIGNALING_VIA_NFKB
hsa-miR-148a-3p	PPARGC1A	TargetScan, miRNAorg, PITA, PicTar	HYPOXIA
hsa-miR-148a-3p	PTGER3	miRNAorg, PITA, miRDB	ESTROGEN_RESPONSE_LATE
hsa-miR-148a-3p	SGCB	TargetScan, miRNAorg, PITA, miRDB	EPITHELIAL_MESENCHYMAL_TRANSITION
hsa-miR-148a-3p	SOCS3	TargetScan, PITA	TNFA_SIGNALING_VIA_NFKB
hsa-miR-182-5p	ABCB1	miRNAorg, MicrocosmTargets, PITA	IL2_STAT5_SIGNALING, KRAS_SIGNALING_UP
hsa-miR-182-5p	ABLIM1	TargetScan, PITA, PicTar	ESTROGEN_RESPONSE_EARLY, MYOGENESIS
hsa-miR-182-5p	CACNA2D2	TargetScan, PITA, PicTar	ESTROGEN_RESPONSE_LATE
hsa-miR-182-5p	CALB2	miRNAorg, MicrocosmTargets, PITA	ESTROGEN_RESPONSE_EARLY
hsa-miR-182-5p	CBFA2T3	TargetScan, miRNAorg, PITA, PicTar, miRDB	ESTROGEN_RESPONSE_EARLY
hsa-miR-182-5p	CD69	miRNAorg, PITA, miRDB	TNFA_SIGNALING_VIA_NFKB, INFLAMMATORY_RESPONSE, APOPTOSIS
hsa-miR-182-5p	DUSP1	TargetScan, miRNAorg, PITA	TNFA_SIGNALING_VIA_NFKB, HYPOXIA
hsa-miR-182-5p	EGR3	TargetScan, miRNAorg, PITA, PicTar, miRDB	TNFA_SIGNALING_VIA_NFKB, APOPTOSIS, ESTROGEN_RESPONSE_EARLY, ESTROGEN_RESPONSE_LATE
hsa-miR-182-5p	FAM134B	TargetScan, miRNAorg, PITA, PicTar, miRDB	ESTROGEN_RESPONSE_EARLY
hsa-miR-182-5p	GPC3	TargetScan, miRNAorg, PITA, PicTar	INFLAMMATORY_RESPONSE, HYPOXIA
hsa-miR-182-5p	HBEGF	TargetScan, miRNAorg, PITA, PicTar	TNFA_SIGNALING_VIA_NFKB, INFLAMMATORY_RESPONSE, KRAS_SIGNALING_UP, MYOGENESIS
hsa-miR-182-5p	LDB3	TargetScan, miRNAorg, PITA, miRDB	MYOGENESIS
hsa-miR-182-5p	MATN3	miRNAorg, MicrocosmTargets, PITA	EPITHELIAL_MESENCHYMAL_TRANSITION
hsa-miR-182-5p	PDPN	TargetScan, miRNAorg, MicrocosmTargets, PITA	INFLAMMATORY_RESPONSE
hsa-miR-182-5p	PTGER3	TargetScan, miRNAorg, MicrocosmTargets, PITA, PicTar	ESTROGEN_RESPONSE_LATE
hsa-miR-182-5p	REEP1	TargetScan, PITA, PicTar	ESTROGEN_RESPONSE_EARLY, MYOGENESIS
hsa-miR-182-5p	RNF144B	miRNAorg, PITA, PicTar	INFLAMMATORY_RESPONSE
hsa-miR-182-5p	SH3BP5	TargetScan, miRNAorg, PITA	ESTROGEN_RESPONSE_EARLY
hsa-miR-182-5p	SIK1	TargetScan, PITA, PicTar	TNFA_SIGNALING_VIA_NFKB
hsa-miR-182-5p	SLC1A1	TargetScan, miRNAorg, PITA, PicTar	ESTROGEN_RESPONSE_EARLY
hsa-miR-182-5p	SLC2A3	miRNAorg, PITA, miRDB	TNFA_SIGNALING_VIA_NFKB, IL2_STAT5_SIGNALING, HYPOXIA
hsa-miR-182-5p	VLDLR	TargetScan, miRNAorg, MicrocosmTargets, PITA, PicTar, miRDB	HYPOXIA
hsa-miR-182-5p	ZFP36	TargetScan, miRNAorg, MicrocosmTargets, PITA, PicTar	TNFA_SIGNALING_VIA_NFKB, HYPOXIA, ESTROGEN_RESPONSE_LATE
hsa-miR-183-5p	CACNA2D2	TargetScan, miRNAorg	ESTROGEN_RESPONSE_LATE
hsa-miR-183-5p	CCL4	miRNAorg, MicrocosmTargets, PITA	TNFA_SIGNALING_VIA_NFKB
hsa-miR-183-5p	CX3CL1	TargetScan, miRNAorg, PITA	INFLAMMATORY_RESPONSE
hsa-miR-183-5p	FBLN1	miRNAorg, MicrocosmTargets, PITA	EPITHELIAL_MESENCHYMAL_TRANSITION
hsa-miR-183-5p	FHL1	TargetScan	MYOGENESIS
hsa-miR-183-5p	GJA5	TargetScan, PITA	MYOGENESIS
hsa-miR-183-5p	HBEGF	miRNAorg, MicrocosmTargets, PITA, PicTar	TNFA_SIGNALING_VIA_NFKB, INFLAMMATORY_RESPONSE, KRAS_SIGNALING_UP, MYOGENESIS

hsa-miR-183-5p	NDST1	TargetScan, PITA	HYPOXIA
hsa-miR-183-5p	NR4A2	TargetScan, miRNAorg, PITA, PicTar	TNFA_SIGNALING_VIA_NFKB
hsa-miR-183-5p	NR4A3	TargetScan	TNFA_SIGNALING_VIA_NFKB
hsa-miR-183-5p	PCOLCE2	miRNAorg, MicrocosmTargets, PITA	EPITHELIAL_MESENCHYMAL_TRANSITION
hsa-miR-183-5p	PLAGL1	TargetScan	IL2_STAT5_SIGNALING
hsa-miR-183-5p	PTCH1	TargetScan, miRNAorg, PITA	IL2_STAT5_SIGNALING
hsa-miR-183-5p	PTGER3	TargetScan, PITA	ESTROGEN_RESPONSE_LATE
hsa-miR-183-5p	RHOB	TargetScan, PicTar	TNFA_SIGNALING_VIA_NFKB, EPITHELIAL_MESENCHYMAL_TRANSITION, IL2_STAT5_SIGNALING, APOPTOSIS
hsa-miR-183-5p	SH3BP5	TargetScan	ESTROGEN_RESPONSE_EARLY
hsa-miR-183-5p	SLC1A1	TargetScan, PITA	ESTROGEN_RESPONSE_EARLY
hsa-miR-183-5p	STC1	TargetScan, miRNAorg, PITA, PicTar, miRDB	HYPOXIA
hsa-miR-183-5p	VLDLR	miRNAorg, MicrocosmTargets, PITA	HYPOXIA
hsa-miR-200b-3p	APOLD1	miRNAorg, MicrocosmTargets, PITA	TNFA_SIGNALING_VIA_NFKB, IL2_STAT5_SIGNALING, KRAS_SIGNALING_UP, APOPTOSIS
hsa-miR-200b-3p	CFL2	TargetScan, miRNAorg, PITA, PicTar, miRDB	TNFA_SIGNALING_VIA_NFKB, INFLAMMATORY_RESPONSE, IL2_STAT5_SIGNALING, HYPOXIA
hsa-miR-200b-3p	CSF3R	miRNAorg, MicrocosmTargets, PITA, miRDB	TNFA_SIGNALING_VIA_NFKB
hsa-miR-200b-3p	CTNND2	TargetScan, miRNAorg, PITA, PicTar, miRDB	TNFA_SIGNALING_VIA_NFKB
hsa-miR-200b-3p	EDNRA	miRNAorg, PITA, PicTar	IL2_STAT5_SIGNALING
hsa-miR-200b-3p	EFNB2	TargetScan, miRNAorg, PITA, PicTar	IL2_STAT5_SIGNALING
hsa-miR-200b-3p	FOXF2	TargetScan, miRNAorg, PITA	EPITHELIAL_MESENCHYMAL_TRANSITION
hsa-miR-200b-3p	GPR146	TargetScan, miRNAorg, PITA, PicTar	INFLAMMATORY_RESPONSE
hsa-miR-200b-3p	ITGA1	miRNAorg, PITA, miRDB	TNFA_SIGNALING_VIA_NFKB, HYPOXIA
hsa-miR-200b-3p	KIAA1462	TargetScan, miRNAorg, PITA	TNFA_SIGNALING_VIA_NFKB, APOPTOSIS, ESTROGEN_RESPONSE_EARLY, ESTROGEN_RESPONSE_LATE
hsa-miR-200b-3p	KLF4	TargetScan, miRNAorg, PITA, PicTar, miRDB	MYOGENESIS
hsa-miR-200b-3p	LEPR	miRNAorg, PITA, PicTar, miRDB	KRAS_SIGNALING_UP
hsa-miR-200b-3p	NR5A2	TargetScan, miRNAorg, PITA, PicTar, miRDB	TNFA_SIGNALING_VIA_NFKB, EPITHELIAL_MESENCHYMAL_TRANSITION, KRAS_SIGNALING_UP, ESTROGEN_RESPONSE_LATE
hsa-miR-200b-3p	PKHD1L1	miRNAorg, PITA, miRDB	TNFA_SIGNALING_VIA_NFKB, KRAS_SIGNALING_UP, ESTROGEN_RESPONSE_EARLY, ESTROGEN_RESPONSE_LATE
hsa-miR-200b-3p	PKIA	TargetScan, miRNAorg, PITA, PicTar	TNFA_SIGNALING_VIA_NFKB, INFLAMMATORY_RESPONSE, IL2_STAT5_SIGNALING, HYPOXIA
hsa-miR-200b-3p	PLK2	TargetScan, miRNAorg, MicrocosmTargets, PITA, PicTar, miRDB	TNFA_SIGNALING_VIA_NFKB
hsa-miR-200b-3p	RELN	TargetScan, miRNAorg, MicrocosmTargets, PITA, miRDB	EPITHELIAL_MESENCHYMAL_TRANSITION, MYOGENESIS
hsa-miR-200b-3p	RTKN2	miRNAorg, PITA, miRDB	HYPOXIA
hsa-miR-200b-3p	SNAI2	miRNAorg, PITA, miRDB	MYOGENESIS
hsa-miR-200b-3p	SRGAP1	TargetScan, miRNAorg, PITA, miRDB	TNFA_SIGNALING_VIA_NFKB
hsa-miR-200b-3p	ST6GALNAC5	TargetScan, miRNAorg, MicrocosmTargets, PITA, PicTar	TNFA_SIGNALING_VIA_NFKB
hsa-miR-200b-3p	STARD13	TargetScan, miRNAorg, PITA, miRDB	IL2_STAT5_SIGNALING
hsa-miR-200b-3p	TBX5	TargetScan, miRNAorg, PITA, miRDB	ESTROGEN_RESPONSE_EARLY, MYOGENESIS
hsa-miR-200b-3p	TCF4	TargetScan, miRNAorg, MicrocosmTargets, PITA	KRAS_SIGNALING_UP

hsa-miR-200b-3p	VLDLR	TargetScan, miRNAorg, MicrocosmTargets, PITA, PicTar, miRDB	MYOGENESIS
hsa-miR-200b-3p	WIF1	miRNAorg, MicrocosmTargets, PITA, miRDB	MYOGENESIS
hsa-miR-204-5p	ATP10B	miRNAorg, PITA, miRDB	IL2_STAT5_SIGNALING
hsa-miR-204-5p	AURKB	miRNAorg, PITA	EPITHELIAL_MESENCHYMAL_TRANSITION
hsa-miR-204-5p	BCL11A	TargetScan, PITA, PicTar	EPITHELIAL_MESENCHYMAL_TRANSITION
hsa-miR-204-5p	FERMT1	miRNAorg, PITA, miRDB	KRAS_SIGNALING_UP
hsa-miR-204-5p	GPT2	TargetScan, miRNAorg, PITA	HYPOXIA
hsa-miR-204-5p	RAMP1	miRNAorg, MicrocosmTargets, PITA	TNFA_SIGNALING_VIA_NFKB, IL2_STAT5_SIGNALING, KRAS_SIGNALING_UP, APOPTOSIS
hsa-miR-204-5p	TRIM2	TargetScan, PITA, PicTar	TNFA_SIGNALING_VIA_NFKB, INFLAMMATORY_RESPONSE, IL2_STAT5_SIGNALING, HYPOXIA
hsa-miR-21-3p	ANGPTL7	TargetScan, miRDB	TNFA_SIGNALING_VIA_NFKB
hsa-miR-21-3p	BMP6	TargetScan, MicrocosmTargets, miRDB	TNFA_SIGNALING_VIA_NFKB
hsa-miR-21-3p	CHRDL1	TargetScan, MicrocosmTargets	EPITHELIAL_MESENCHYMAL_TRANSITION
hsa-miR-21-3p	CHRM3	TargetScan	GLYCOLYSIS
hsa-miR-21-3p	FAM124B	TargetScan	E2F_TARGETS, G2M_CHECKPOINT
hsa-miR-21-3p	GALNT15	TargetScan	EPITHELIAL_MESENCHYMAL_TRANSITION, GLYCOLYSIS
hsa-miR-21-3p	GFRA1	TargetScan, miRDB	G2M_CHECKPOINT
hsa-miR-21-3p	GPR146	TargetScan, MicrocosmTargets, miRDB	KRAS_SIGNALING_UP
hsa-miR-21-3p	IGFBP6	TargetScan, MicrocosmTargets	EPITHELIAL_MESENCHYMAL_TRANSITION
hsa-miR-21-3p	MATN3	TargetScan	EPITHELIAL_MESENCHYMAL_TRANSITION
hsa-miR-21-3p	NTN4	TargetScan	KRAS_SIGNALING_UP
hsa-miR-21-3p	VAMP5	TargetScan, MicrocosmTargets	INFLAMMATORY_RESPONSE
hsa-miR-21-5p	ALDH1A1	miRNAorg, MicrocosmTargets, PITA, miRDB	TNFA_SIGNALING_VIA_NFKB
hsa-miR-21-5p	EGR3	TargetScan, miRNAorg, PITA	ESTROGEN_RESPONSE_EARLY
hsa-miR-21-5p	FRMD3	miRNAorg, MicrocosmTargets, PITA, miRDB	APOPTOSIS
hsa-miR-21-5p	GIPC3	miRNAorg, PITA, miRDB	IL2_STAT5_SIGNALING
hsa-miR-21-5p	KLF6	TargetScan, miRNAorg, miRDB	EPITHELIAL_MESENCHYMAL_TRANSITION
hsa-miR-21-5p	NEGR1	miRNAorg, MicrocosmTargets, PITA, miRDB	EPITHELIAL_MESENCHYMAL_TRANSITION
hsa-miR-21-5p	PDZD2	TargetScan, miRNAorg, PITA, PicTar, miRDB	KRAS_SIGNALING_UP
hsa-miR-21-5p	RTKN2	miRNAorg, PITA, miRDB	ESTROGEN_RESPONSE_EARLY, MYOGENESIS
hsa-miR-21-5p	SETBP1	miRNAorg, MicrocosmTargets, PITA	TNFA_SIGNALING_VIA_NFKB, EPITHELIAL_MESENCHYMAL_TRANSITION, IL2_STAT5_SIGNALING, APOPTOSIS
hsa-miR-21-5p	VSNL1	miRNAorg, MicrocosmTargets, PITA	IL2_STAT5_SIGNALING
hsa-miR-218-1-3p	COL28A1	TargetScan	INFLAMMATORY_RESPONSE
hsa-miR-218-1-3p	KIF4A	TargetScan	TNFA_SIGNALING_VIA_NFKB, INFLAMMATORY_RESPONSE, APOPTOSIS
hsa-miR-218-1-3p	PTGES	TargetScan, MicrocosmTargets	TNFA_SIGNALING_VIA_NFKB, APOPTOSIS, ESTROGEN_RESPONSE_EARLY, ESTROGEN_RESPONSE_LATE
hsa-miR-218-5p	BCL11A	TargetScan, miRNAorg, PITA	TNFA_SIGNALING_VIA_NFKB, INFLAMMATORY_RESPONSE, IL2_STAT5_SIGNALING, HYPOXIA
hsa-miR-218-5p	CLEC5A	miRNAorg, PITA, miRDB	EPITHELIAL_MESENCHYMAL_TRANSITION
hsa-miR-218-5p	COL1A1	TargetScan, miRNAorg, PITA, PicTar, miRDB	EPITHELIAL_MESENCHYMAL_TRANSITION
hsa-miR-218-5p	EGLN3	TargetScan, miRNAorg, PITA, miRDB	ESTROGEN_RESPONSE_LATE
hsa-miR-218-5p	LIPG	TargetScan, miRNAorg, PITA	EPITHELIAL_MESENCHYMAL_TRANSITION
hsa-miR-218-5p	MACC1	miRNAorg, PITA, miRDB	E2F_TARGETS
hsa-miR-218-5p	RAB3B	miRNAorg, PITA, miRDB	G2M_CHECKPOINT
hsa-miR-218-5p	SLC12A2	TargetScan, miRNAorg, PITA, PicTar, miRDB	ESTROGEN_RESPONSE_EARLY, P53_PATHWAY

hsa-miR-218-5p	SLC5A3	TargetScan, PITA, miRDB	E2F_TARGETS, G2M_CHECKPOINT, MITOTIC_SPINDLE
hsa-miR-218-5p	TMEM156	TargetScan, PITA	ESTROGEN_RESPONSE_EARLY, ESTROGEN_RESPONSE_LATE
hsa-miR-223-3p	ATP1B1	TargetScan, miRNAorg, MicrocosmTargets, PITA, PicTar	MITOTIC_SPINDLE
hsa-miR-223-3p	FCRL5	miRNAorg, MicrocosmTargets, PITA	EPITHELIAL_MESENCHYMAL_TRANSITION
hsa-miR-223-3p	HOOK1	miRNAorg, PITA, miRDB	GLYCOLYSIS
hsa-miR-223-3p	PAX9	miRNAorg, MicrocosmTargets, PITA, miRDB	ESTROGEN_RESPONSE_EARLY, ESTROGEN_RESPONSE_LATE
hsa-miR-223-3p	PLCH1	miRNAorg, MicrocosmTargets, PITA	KRAS_SIGNALING_UP
hsa-miR-223-3p	RALGPS2	TargetScan, miRNAorg, PITA, PicTar	EPITHELIAL_MESENCHYMAL_TRANSITION
hsa-miR-223-3p	ST6GALNAC1	miRNAorg, MicrocosmTargets, PITA	ESTROGEN_RESPONSE_EARLY, P53_PATHWAY
hsa-miR-223-5p	GPX8	TargetScan	G2M_CHECKPOINT
hsa-miR-223-5p	MACC1	TargetScan, miRDB	P53_PATHWAY
hsa-miR-223-5p	PLEKHS1	TargetScan	EPITHELIAL_MESENCHYMAL_TRANSITION
hsa-miR-27a-5p	FAM83A	TargetScan	GLYCOLYSIS
hsa-miR-27a-5p	FCRL5	TargetScan	ESTROGEN_RESPONSE_EARLY
hsa-miR-27a-5p	HOXB7	TargetScan, MicrocosmTargets	ESTROGEN_RESPONSE_EARLY, P53_PATHWAY
hsa-miR-27a-5p	IGF2BP3	TargetScan	ESTROGEN_RESPONSE_LATE
hsa-miR-27a-5p	KLHDC7A	TargetScan	E2F_TARGETS
hsa-miR-27a-5p	SLC17A9	TargetScan	ESTROGEN_RESPONSE_LATE
hsa-miR-27a-5p	TREM2	TargetScan	ESTROGEN_RESPONSE_EARLY
hsa-miR-2a-5p	CCDC12B	TargetScan	KRAS_SIGNALING_UP
hsa-miR-2a-5p	RGS2	TargetScan, MicrocosmTargets	GLYCOLYSIS
hsa-miR-2b-5p	BEX1	TargetScan, MicrocosmTargets	KRAS_SIGNALING_UP
hsa-miR-2b-5p	MAMDC2	TargetScan, MicrocosmTargets	E2F_TARGETS
hsa-miR-2b-5p	RGS2	TargetScan, MicrocosmTargets	E2F_TARGETS, ESTROGEN_RESPONSE_LATE, G2M_CHECKPOINT, MITOTIC_SPINDLE
hsa-miR-30a-5p	CDCA7	TargetScan, miRNAorg, PITA	TNFA_SIGNALING_VIA_NFKB, INFLAMMATORY_RESPONSE, APOPTOSIS
hsa-miR-30a-5p	CTHRC1	TargetScan, miRNAorg, MicrocosmTargets, PITA	TNFA_SIGNALING_VIA_NFKB, APOPTOSIS, ESTROGEN_RESPONSE_EARLY, ESTROGEN_RESPONSE_LATE
hsa-miR-30a-5p	CYP24A1	TargetScan, miRNAorg, PITA, miRDB	TNFA_SIGNALING_VIA_NFKB, INFLAMMATORY_RESPONSE, HYPOXIA
hsa-miR-30a-5p	DOK5	miRNAorg, MicrocosmTargets, PITA, miRDB	EPITHELIAL_MESENCHYMAL_TRANSITION
hsa-miR-30a-5p	FAM46C	TargetScan, miRNAorg, PITA, PicTar	TNFA_SIGNALING_VIA_NFKB, EPITHELIAL_MESENCHYMAL_TRANSITION, IL2_STAT5_SIGNALING, APOPTOSIS, MYOGENESIS
hsa-miR-30a-5p	HMGB3	TargetScan, PITA, PicTar	HYPOXIA
hsa-miR-30a-5p	PGM2L1	TargetScan, miRNAorg, PITA	ESTROGEN_RESPONSE_EARLY, MYOGENESIS
hsa-miR-30a-5p	RAB15	TargetScan, miRNAorg, PITA, PicTar, miRDB	HYPOXIA
hsa-miR-30a-5p	RASGEF1A	TargetScan, PITA, PicTar	INFLAMMATORY_RESPONSE, IL2_STAT5_SIGNALING, APOPTOSIS
hsa-miR-30a-5p	SLC39A11	TargetScan, PITA, PicTar	EPITHELIAL_MESENCHYMAL_TRANSITION
hsa-miR-30a-5p	ZIC2	miRNAorg, PITA, PicTar	EPITHELIAL_MESENCHYMAL_TRANSITION
hsa-miR-31b-3p	CD69	TargetScan	E2F_TARGETS
hsa-miR-31b-3p	CPEB1	TargetScan	EPITHELIAL_MESENCHYMAL_TRANSITION
hsa-miR-31b-3p	FAM46B	TargetScan	GLYCOLYSIS
hsa-miR-31b-3p	FERMT2	TargetScan	KRAS_SIGNALING_UP

hsa-miR-31b-3p	FOXF2	TargetScan	EPITHELIAL_MESENCHYMAL_TRANSITION
hsa-miR-31b-3p	HECW2	TargetScan	ESTROGEN_RESPONSE_EARLY, ESTROGEN_RESPONSE_LATE
hsa-miR-31b-3p	HEG1	TargetScan	KRAS_SIGNALING_UP
hsa-miR-31b-3p	NDRG2	TargetScan	E2F_TARGETS, G2M_CHECKPOINT
hsa-miR-31b-3p	PDE7B	TargetScan	E2F_TARGETS, G2M_CHECKPOINT, MITOTIC_SPINDLE
hsa-miR-31b-3p	PLLP	TargetScan	EPITHELIAL_MESENCHYMAL_TRANSITION
hsa-miR-338-3p	FCRL2	miRNAorg, MicrocosmTargets, PITA	GLYCOLYSIS, KRAS_SIGNALING_UP
hsa-miR-338-3p	KIAA1199	TargetScan, miRNAorg, MicrocosmTargets, PITA	EPITHELIAL_MESENCHYMAL_TRANSITION
hsa-miR-338-3p	NHS	TargetScan, PITA	ESTROGEN_RESPONSE_EARLY
hsa-miR-338-3p	TBC1D8	TargetScan, miRNAorg, PITA, miRDB	ESTROGEN_RESPONSE_EARLY
hsa-miR-338-5p	BCAT1	TargetScan, PITA, miRDB	GLYCOLYSIS
hsa-miR-338-5p	CDCA8	TargetScan, PITA, miRDB	GLYCOLYSIS
hsa-miR-338-5p	CXCL14	TargetScan, PITA, miRDB	EPITHELIAL_MESENCHYMAL_TRANSITION
hsa-miR-338-5p	HMGB3	TargetScan, PITA, miRDB	KRAS_SIGNALING_UP
hsa-miR-375	C7	TargetScan, miRNAorg, PITA	P53_PATHWAY
hsa-miR-375	FOXF1	TargetScan, miRNAorg, PITA	EPITHELIAL_MESENCHYMAL_TRANSITION
hsa-miR-375	GPR146	miRNAorg, MicrocosmTargets, PITA	GLYCOLYSIS
hsa-miR-375	MEIS1	TargetScan, miRNAorg, PicTar	E2F_TARGETS
hsa-miR-375	RYR2	miRNAorg, MicrocosmTargets, PITA	EPITHELIAL_MESENCHYMAL_TRANSITION
hsa-miR-375	SHANK3	TargetScan, miRNAorg, PITA	E2F_TARGETS, G2M_CHECKPOINT
hsa-miR-3a-3p	DGCR6	TargetScan	ESTROGEN_RESPONSE_EARLY, ESTROGEN_RESPONSE_LATE
hsa-miR-3a-5p	KIAA11	TargetScan, miRNAorg, PITA	EPITHELIAL_MESENCHYMAL_TRANSITION
hsa-miR-3c-2-3p	AQP3	TargetScan	ESTROGEN_RESPONSE_EARLY, GLYCOLYSIS
hsa-miR-3c-2-3p	DLX3	TargetScan	ESTROGEN_RESPONSE_EARLY, ESTROGEN_RESPONSE_LATE
hsa-miR-3c-2-3p	DPP4	TargetScan	ESTROGEN_RESPONSE_LATE
hsa-miR-3c-2-3p	FRMPD3	TargetScan	E2F_TARGETS
hsa-miR-3c-2-3p	GPX2	TargetScan	ESTROGEN_RESPONSE_LATE
hsa-miR-3c-2-3p	IFI6	TargetScan	EPITHELIAL_MESENCHYMAL_TRANSITION
hsa-miR-3c-2-3p	IYD	TargetScan	E2F_TARGETS, G2M_CHECKPOINT
hsa-miR-3c-2-3p	MS4A1	TargetScan	G2M_CHECKPOINT, MITOTIC_SPINDLE
hsa-miR-3c-2-3p	MXRA5	TargetScan	EPITHELIAL_MESENCHYMAL_TRANSITION
hsa-miR-429	AHNAK	TargetScan, miRNAorg, PITA	IL2_STAT5_SIGNALING
hsa-miR-429	AKAP2	TargetScan, miRNAorg, PITA	IL2_STAT5_SIGNALING
hsa-miR-429	CDH6	TargetScan, miRNAorg, PITA	EPITHELIAL_MESENCHYMAL_TRANSITION
hsa-miR-429	CSF3R	miRNAorg, MicrocosmTargets, PITA, miRDB	INFLAMMATORY_RESPONSE
hsa-miR-429	CYR61	miRNAorg, MicrocosmTargets, PITA	TNFA_SIGNALING_VIA_NFKB, EPITHELIAL_MESENCHYMAL_TRANSITION, HYPOXIA
hsa-miR-429	DUSP1	TargetScan, miRNAorg, MicrocosmTargets, PITA, miRDB	TNFA_SIGNALING_VIA_NFKB, HYPOXIA
hsa-miR-429	EGR3	TargetScan, miRNAorg, PITA, miRDB	TNFA_SIGNALING_VIA_NFKB, APOPTOSIS, ESTROGEN_RESPONSE_EARLY, ESTROGEN_RESPONSE_LATE
hsa-miR-429	EMP1	TargetScan, miRNAorg, PITA	IL2_STAT5_SIGNALING, KRAS_SIGNALING_UP, APOPTOSIS
hsa-miR-429	FERMT2	miRNAorg, PITA, miRDB	EPITHELIAL_MESENCHYMAL_TRANSITION
hsa-miR-429	FHL1	TargetScan, miRNAorg, PITA, miRDB	MYOGENESIS

hsa-miR-429	FLT4	miRNAorg, MicrocosmTargets, PITA	KRAS_SIGNALING_UP
hsa-miR-429	GABARAPL 1	miRNAorg, MicrocosmTargets, PITA	IL2_STAT5_SIGNALING
hsa-miR-429	KLF4	TargetScan, miRNAorg, PITA, miRDB	TNFA_SIGNALING_VIA_NFKB, KRAS_SIGNALING_UP, ESTROGEN_RESPONSE_EARLY, ESTROGEN_RESPONSE_LATE
hsa-miR-429	KLF6	TargetScan, miRNAorg	TNFA_SIGNALING_VIA_NFKB, INFLAMMATORY_RESPONSE, IL2_STAT5_SIGNALING, HYPOXIA
hsa-miR-429	KLF9	TargetScan, miRNAorg, PITA	TNFA_SIGNALING_VIA_NFKB
hsa-miR-429	MATN3	miRNAorg, MicrocosmTargets, PITA	EPITHELIAL_MESENCHYMAL_TRANSITION
hsa-miR-429	MYLK	TargetScan, miRNAorg, PITA	EPITHELIAL_MESENCHYMAL_TRANSITION, MYOGENESIS
hsa-miR-429	PKIA	TargetScan, miRNAorg, PITA	MYOGENESIS
hsa-miR-429	PLK2	TargetScan, miRNAorg, PITA, miRDB	TNFA_SIGNALING_VIA_NFKB
hsa-miR-429	PPAP2B	TargetScan, miRNAorg, PITA	TNFA_SIGNALING_VIA_NFKB
hsa-miR-429	PTCH1	TargetScan, miRNAorg, PITA	IL2_STAT5_SIGNALING
hsa-miR-429	REEP1	TargetScan, miRNAorg, PITA, miRDB	ESTROGEN_RESPONSE_EARLY, MYOGENESIS
hsa-miR-429	RELN	TargetScan, miRNAorg, MicrocosmTargets, PITA, miRDB	KRAS_SIGNALING_UP
hsa-miR-429	SCD	TargetScan, miRNAorg, miRDB	MYOGENESIS
hsa-miR-429	SCHIP1	TargetScan, miRNAorg, MicrocosmTargets, PITA, miRDB	MYOGENESIS
hsa-miR-429	SLC39A8	miRNAorg, PITA, miRDB	IL2_STAT5_SIGNALING
hsa-miR-429	SLIT2	TargetScan	EPITHELIAL_MESENCHYMAL_TRANSITION
hsa-miR-429	SNAI2	miRNAorg, PITA, miRDB	EPITHELIAL_MESENCHYMAL_TRANSITION
hsa-miR-429	TMEM100	miRNAorg, MicrocosmTargets, PITA	KRAS_SIGNALING_UP
hsa-miR-429	VLDLR	TargetScan, miRNAorg, MicrocosmTargets, PITA, miRDB	HYPOXIA
hsa-miR-429	ZFP36	miRNAorg, MicrocosmTargets, PITA	TNFA_SIGNALING_VIA_NFKB, HYPOXIA, ESTROGEN_RESPONSE_LATE
hsa-miR-450b-5p	ABLIM1	TargetScan, PITA, miRDB	ESTROGEN_RESPONSE_EARLY, MYOGENESIS
hsa-miR-450b-5p	CAMK2N1	TargetScan, MicrocosmTargets, PITA, miRDB	INFLAMMATORY_RESPONSE
hsa-miR-450b-5p	CLEC3B	TargetScan, MicrocosmTargets, PITA	TNFA_SIGNALING_VIA_NFKB
hsa-miR-450b-5p	GPM6A	TargetScan, PITA, miRDB	ESTROGEN_RESPONSE_EARLY
hsa-miR-450b-5p	HSD11B1	miRNAorg, PITA, miRDB	EPITHELIAL_MESENCHYMAL_TRANSITION
hsa-miR-450b-5p	PCDH17	TargetScan, PITA, miRDB	INFLAMMATORY_RESPONSE, HYPOXIA
hsa-miR-450b-5p	RBMS3	TargetScan, PITA, miRDB	KRAS_SIGNALING_UP
hsa-miR-450b-5p	TCEAL7	miRNAorg, PITA, miRDB	ESTROGEN_RESPONSE_EARLY, MYOGENESIS, ESTROGEN_RESPONSE_LATE
hsa-miR-45b-5p	CLEC12A	TargetScan	TNFA_SIGNALING_VIA_NFKB
hsa-miR-45b-5p	PIK3R1	TargetScan, PITA	EPITHELIAL_MESENCHYMAL_TRANSITION
hsa-miR-45b-5p	PIP5K1B	TargetScan, PITA, miRDB	KRAS_SIGNALING_UP
hsa-miR-45b-5p	RGS5	TargetScan, PITA	EPITHELIAL_MESENCHYMAL_TRANSITION, APOPTOSIS
hsa-miR-45b-5p	SPARCL1	TargetScan, PITA	EPITHELIAL_MESENCHYMAL_TRANSITION
hsa-miR-45b-5p	VEGFC	TargetScan, PITA	GLYCOLYSIS
hsa-miR-486-3p	AQP3	TargetScan, PITA	ESTROGEN_RESPONSE_EARLY
hsa-miR-486-3p	AURKB	TargetScan	E2F_TARGETS, G2M_CHECKPOINT
hsa-miR-486-3p	CDKN2A	TargetScan, PITA	E2F_TARGETS, P53_PATHWAY

hsa-miR-486-3p	CENPM	TargetScan, MicrocosmTargets, PITA	E2F_TARGETS
hsa-miR-486-3p	CLDN9	TargetScan, MicrocosmTargets, PITA	GLYCOLYSIS
hsa-miR-486-3p	CXCL14	TargetScan, PITA	ESTROGEN_RESPONSE_LATE
hsa-miR-486-3p	EPHB2	TargetScan, MicrocosmTargets, PITA	KRAS_SIGNALING_UP
hsa-miR-486-3p	ETV4	TargetScan, PITA	KRAS_SIGNALING_UP
hsa-miR-486-3p	GLS2	TargetScan, PITA	P53_PATHWAY
hsa-miR-486-3p	KCNK5	TargetScan, PITA	ESTROGEN_RESPONSE_EARLY, ESTROGEN_RESPONSE_LATE
hsa-miR-486-3p	KRT17	TargetScan, PITA	P53_PATHWAY
hsa-miR-486-3p	MMP14	TargetScan, PITA	EPITHELIAL_MESENCHYMAL_TRANSITION
hsa-miR-486-3p	PFKP	TargetScan, MicrocosmTargets, PITA	GLYCOLYSIS
hsa-miR-486-3p	PPFIA4	TargetScan, PITA	GLYCOLYSIS
hsa-miR-486-3p	SLC25A1	TargetScan, PITA	GLYCOLYSIS
hsa-miR-486-3p	SLC7A5	TargetScan, PITA	ESTROGEN_RESPONSE_EARLY, ESTROGEN_RESPONSE_LATE, G2M_CHECKPOINT
hsa-miR-486-3p	STEAP3	TargetScan, PITA	P53_PATHWAY
hsa-miR-486-3p	TK1	TargetScan	E2F_TARGETS
hsa-miR-486-5p	BUB1B	miRNAorg, MicrocosmTargets, PITA	E2F_TARGETS
hsa-miR-577	CAV1	TargetScan, PITA, miRDB	HYPOXIA, APOPTOSIS, ESTROGEN_RESPONSE_LATE
hsa-miR-577	CSF3	TargetScan, PITA	INFLAMMATORY_RESPONSE
hsa-miR-577	KLF4	TargetScan, MicrocosmTargets, PITA, miRDB	TNFA_SIGNALING_VIA_NFKB, KRAS_SIGNALING_UP, ESTROGEN_RESPONSE_EARLY, ESTROGEN_RESPONSE_LATE
hsa-miR-577	KLF9	TargetScan, PITA, miRDB	TNFA_SIGNALING_VIA_NFKB
hsa-miR-577	RBP4	TargetScan, miRDB	KRAS_SIGNALING_UP
hsa-miR-577	TGFBR3	TargetScan, PITA, miRDB	EPITHELIAL_MESENCHYMAL_TRANSITION, APOPTOSIS
hsa-miR-584-5p	ADAMDEC1	TargetScan, MicrocosmTargets, PITA	KRAS_SIGNALING_UP
hsa-miR-584-5p	ITGBL1	TargetScan, PITA	KRAS_SIGNALING_UP
hsa-miR-9-5p	ACTN2	TargetScan, miRNAorg, PITA	MYOGENESIS
hsa-miR-9-5p	ANO1	TargetScan, miRNAorg, PITA, PicTar, miRDB	KRAS_SIGNALING_UP
hsa-miR-9-5p	BTG2	TargetScan, miRNAorg, PITA, PicTar	TNFA_SIGNALING_VIA_NFKB, INFLAMMATORY_RESPONSE, APOPTOSIS
hsa-miR-9-5p	CALB2	TargetScan, miRNAorg, MicrocosmTargets, PITA, PicTar, miRDB	ESTROGEN_RESPONSE_EARLY
hsa-miR-9-5p	CCL4	miRNAorg, MicrocosmTargets, PITA	TNFA_SIGNALING_VIA_NFKB
hsa-miR-9-5p	EGR3	TargetScan, PITA, PicTar	TNFA_SIGNALING_VIA_NFKB, APOPTOSIS, ESTROGEN_RESPONSE_EARLY, ESTROGEN_RESPONSE_LATE
hsa-miR-9-5p	IL33	miRNAorg, PITA, miRDB	KRAS_SIGNALING_UP
hsa-miR-9-5p	PMP22	miRNAorg, MicrocosmTargets, PITA, PicTar, miRDB	EPITHELIAL_MESENCHYMAL_TRANSITION
hsa-miR-9-5p	PTCH1	TargetScan, PITA, PicTar	IL2_STAT5_SIGNALING
hsa-miR-9-5p	SELP	miRNAorg, MicrocosmTargets, PITA, miRDB	IL2_STAT5_SIGNALING
hsa-miR-9-5p	SIK1	TargetScan, PITA, PicTar, miRDB	TNFA_SIGNALING_VIA_NFKB
hsa-miR-9-5p	SLC1A1	TargetScan, miRNAorg, PITA, PicTar	ESTROGEN_RESPONSE_EARLY
hsa-miR-9-5p	SLC26A2	TargetScan, PITA, miRDB	ESTROGEN_RESPONSE_EARLY, ESTROGEN_RESPONSE_LATE
hsa-miR-9-5p	STC1	miRNAorg, PITA, PicTar	HYPOXIA
hsa-miR-9-5p	VIP	miRNAorg, MicrocosmTargets, PITA	INFLAMMATORY_RESPONSE
hsa-miR-96-5p	AHNAK	miRNAorg, PITA, PicTar	IL2_STAT5_SIGNALING

hsa-miR-96-5p	CACNA2D2	TargetScan, miRNAorg, PITA, PicTar, miRDB	ESTROGEN_RESPONSE_LATE
hsa-miR-96-5p	CADM1	TargetScan, miRNAorg, PITA	EPITHELIAL_MESENCHYMAL_TRANSITION
hsa-miR-96-5p	CALCRL	miRNAorg, PITA, miRDB	INFLAMMATORY_RESPONSE
hsa-miR-96-5p	CAV1	miRNAorg, PITA, PicTar	HYPOXIA, APOPTOSIS, ESTROGEN_RESPONSE_LATE
hsa-miR-96-5p	CBFA2T3	TargetScan, PITA, PicTar	ESTROGEN_RESPONSE_EARLY
hsa-miR-96-5p	CCNA1	miRNAorg, MicrocosmTargets, PITA	APOPTOSIS, ESTROGEN_RESPONSE_LATE
hsa-miR-96-5p	EGR3	TargetScan, miRNAorg, PITA, PicTar	TNFA_SIGNALING_VIA_NFKB, APOPTOSIS, ESTROGEN_RESPONSE_EARLY, ESTROGEN_RESPONSE_LATE
hsa-miR-96-5p	ELTD1	miRNAorg, PITA, miRDB	KRAS_SIGNALING_UP
hsa-miR-96-5p	FAM134B	TargetScan, PITA, PicTar	ESTROGEN_RESPONSE_EARLY
hsa-miR-96-5p	FHL1	TargetScan, miRNAorg, PITA	MYOGENESIS
hsa-miR-96-5p	GFRA1	TargetScan, miRNAorg, PITA	ESTROGEN_RESPONSE_EARLY
hsa-miR-96-5p	GJA5	TargetScan, miRNAorg, PITA	MYOGENESIS
hsa-miR-96-5p	GPC3	TargetScan, miRNAorg, MicrocosmTargets, PITA, PicTar, miRDB	INFLAMMATORY_RESPONSE, HYPOXIA
hsa-miR-96-5p	HBEGF	TargetScan, miRNAorg, MicrocosmTargets, PITA, PicTar, miRDB	TNFA_SIGNALING_VIA_NFKB, INFLAMMATORY_RESPONSE, KRAS_SIGNALING_UP, MYOGENESIS
hsa-miR-96-5p	LDB3	TargetScan, miRNAorg, MicrocosmTargets, PITA	MYOGENESIS
hsa-miR-96-5p	MT2A	TargetScan	HYPOXIA
hsa-miR-96-5p	PLAGL1	TargetScan, miRNAorg, PITA, PicTar	IL2_STAT5_SIGNALING
hsa-miR-96-5p	PTGER3	TargetScan, miRNAorg, PITA, PicTar, miRDB	ESTROGEN_RESPONSE_LATE
hsa-miR-96-5p	REEP1	TargetScan, PITA, PicTar	ESTROGEN_RESPONSE_EARLY, MYOGENESIS
hsa-miR-96-5p	SH3BP5	TargetScan, miRNAorg, PITA, PicTar, miRDB	ESTROGEN_RESPONSE_EARLY
hsa-miR-96-5p	SIK1	TargetScan, PITA, PicTar	TNFA_SIGNALING_VIA_NFKB
hsa-miR-96-5p	TEAD4	miRNAorg, MicrocosmTargets, PITA	MYOGENESIS
hsa-miR-96-5p	VLDLR	TargetScan, miRNAorg, PITA, PicTar	HYPOXIA
hsa-miR-96-5p	ZFP36	miRNAorg, PITA, PicTar	TNFA_SIGNALING_VIA_NFKB, HYPOXIA, ESTROGEN_RESPONSE_LATE

Table S6. List of the 14 candidate miRNAs among the 26 down-regulated DEmiRs from the ES_Korea data set based on the fold change ratio, average expression level, and literature evidences.

DEmiR ID		Dataset								Filtering criteria		Final selection for experiment
		ES_Korea (n=48)				TCGA_LUAD (n=39)				AveExpr>10 or logFC<-2	Manual curation	
		log_2FC	AveExpr	No. of patients		log_2FC	AveExpr	No. of patients				
				Down	Up			Down	Up			
1	hsa-miR-126-3p	-2.0	12.4	49	0	-1.1	10.5	34	4	AveExpr		v
2	hsa-miR-126-5p	-1.9	14.1	49	0	-1.7	8.0	38	0	AveExpr		v
3	hsa-miR-133a-3p	-1.8	6.9	46	3	-2.2	2.9	39	0			
4	hsa-miR-135a-5p	-2.3	3.0	48	1	-1.1	1.5	36	3	logFC	Known	
5	hsa-miR-138-5p	-2.1	5.7	48	1	-1.7	2.5	36	3	logFC	Known	
6	hsa-miR-139-5p	-2.4	5.6	49	0	-1.7	5.1	37	2	logFC		v
7	hsa-miR-1-3p	-1.2	3.1	44	5	-2.9	4.2	39	0			
8	hsa-miR-144-3p	-2.3	8.7	46	3	-2.7	4.5	38	1	logFC	Known	
9	hsa-miR-144-5p	-2.4	8.4	45	4	-3.3	7.7	39	0	logFC		v
10	hsa-miR-145-3p	-1.5	8.2	47	2	-1.7	5.6	39	0			
11	hsa-miR-204-5p	-1.9	6.0	46	3	-1.2	2.3	36	3			
12	hsa-miR-218-1-3p	-1.9	5.4	49	0	-1.1	1.6	39	0		Novel	v
13	hsa-miR-218-5p	-1.9	7.8	49	0	-2.4	6.1	39	0			
14	hsa-miR-223-3p	-1.3	8.6	45	3	-1.1	8.2	33	4		Novel	v
15	hsa-miR-223-5p	-1.3	3.7	44	5	-1.1	2.2	36	3			
16	hsa-miR-27a-5p	-1.5	5.4	44	4	-1.5	4.0	36	3		Novel	v
17	hsa-miR-30a-3p	-2.5	9.2	49	0	-2.5	12.8	39	0	logFC		v
18	hsa-miR-30a-5p	-2.1	14.8	49	0	-2.3	14.1	38	0	logFC, AveExpr		v
19	hsa-miR-30c-2-3p	-2.1	5.7	49	0	-2.7	6.0	39	0	logFC		v
20	hsa-miR-338-3p	-2.8	8.6	47	2	-1.7	8.4	37	2	logFC		v
21	hsa-miR-338-5p	-2.3	4.0	46	3	-1.8	4.0	37	2	logFC		v
22	hsa-miR-451a	-2.2	12.3	47	2	-2.9	9.8	38	1	logFC, AveExpr		v
23	hsa-miR-486-3p	-1.5	3.4	45	4	-0.3	1.0	29	8			
24	hsa-miR-486-5p	-2.5	13.0	46	2	-3.1	8.1	39	0	logFC, AveExpr		v
25	hsa-miR-511-5p	-1.1	4.1	41	8	-0.5	4.2	23	16			
26	hsa-miR-584-5p	-1.6	3.7	47	2	-1.5	5.2	36	3			

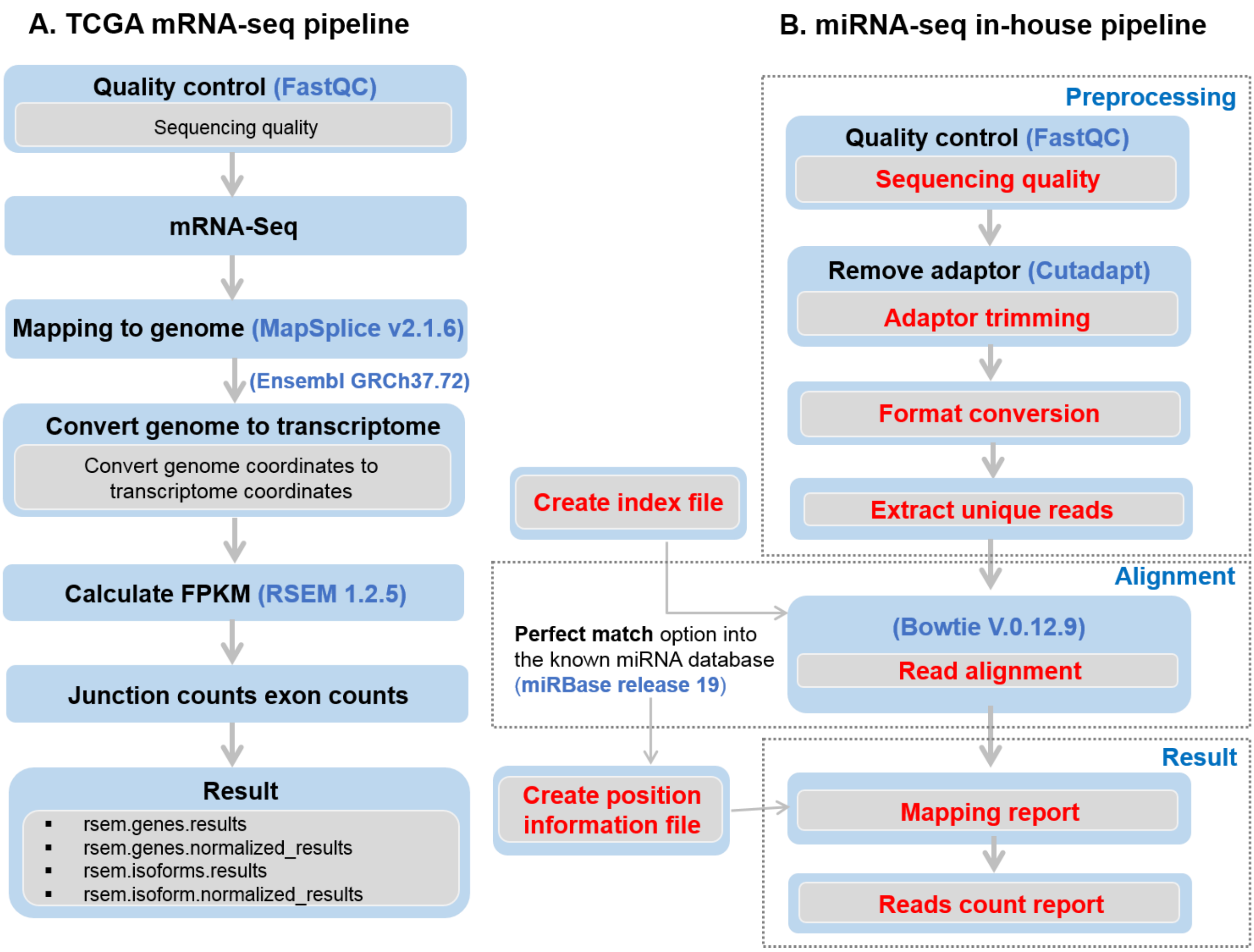

Figure S1. In-house workflows for analyzing miRNA-Seq and RNA-Seq data.

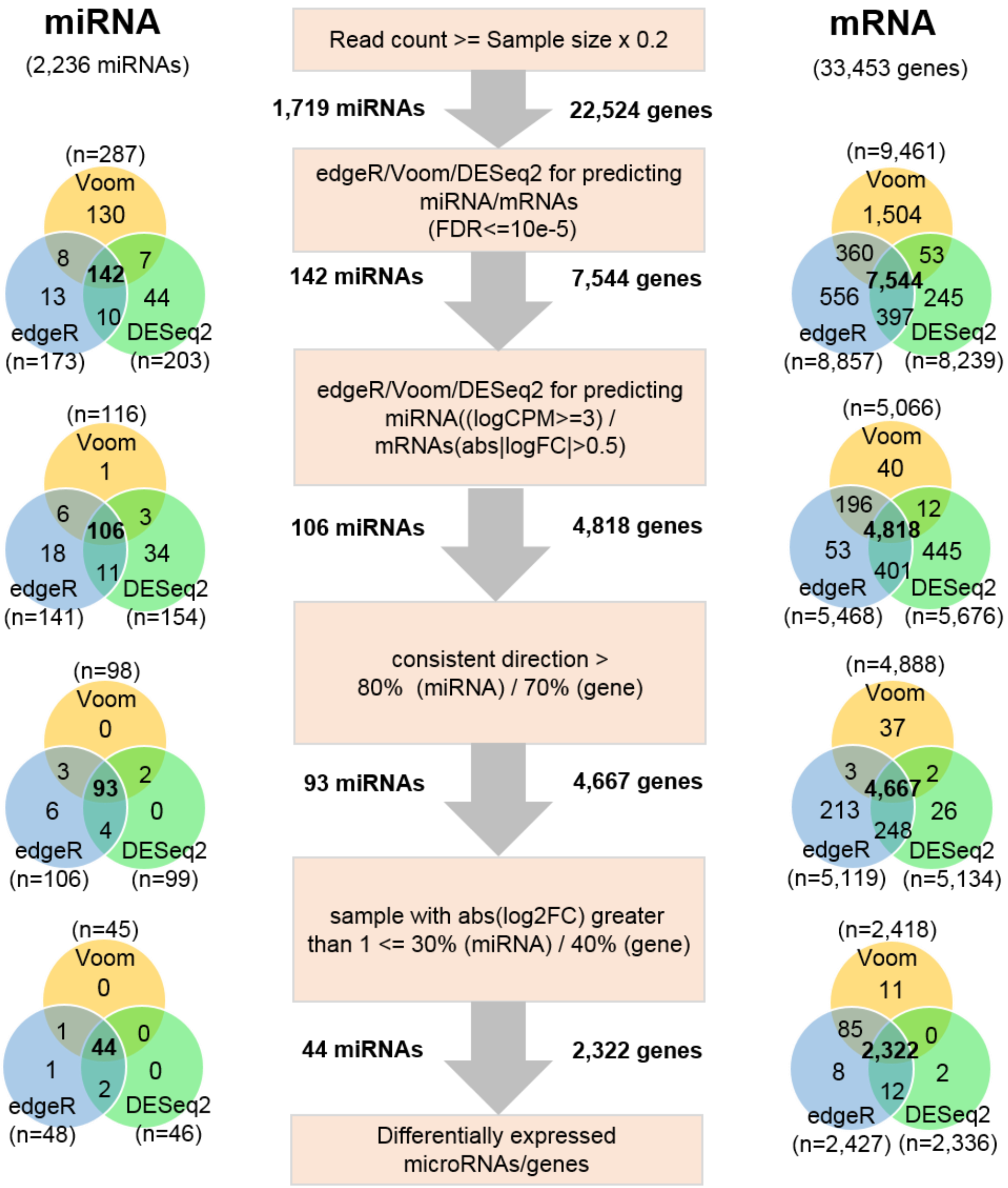

Figure S2. Computational pipeline to identify differentially expressed miRNAs (DEmiRs) and genes (DEGs).

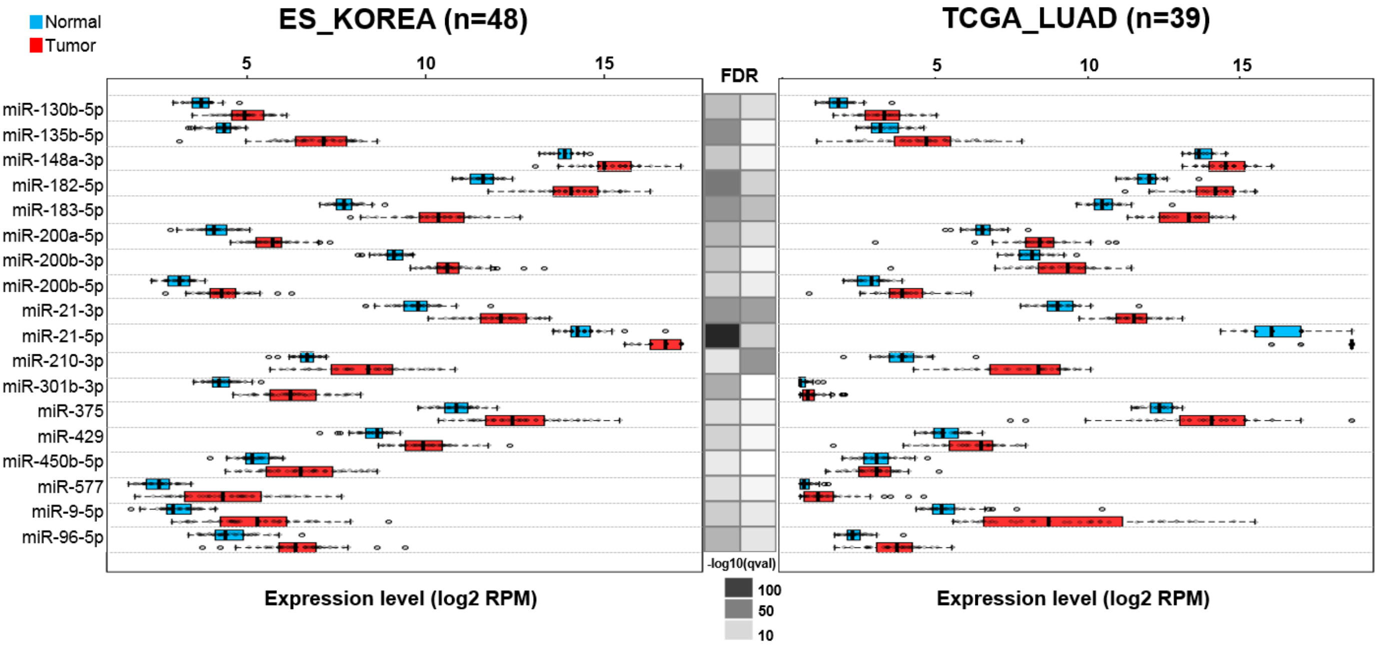

Figure S3. Expression box plots for 18 miRNAs up-regulated in tumor samples of the ES_Korea cohort.
